# Learning anisotropic interaction rules from individual trajectories in a heterogeneous cellular population

**DOI:** 10.1098/rsif.2022.0412

**Published:** 2022-10-12

**Authors:** Daniel A. Messenger, Graycen E. Wheeler, Xuedong Liu, David M. Bortz

**Affiliations:** ^1^ Department of Applied Mathematics, University of Colorado, Boulder, CO 80309-0526, USA; ^2^ Department of Biochemistry, University of Colorado, Boulder, CO 80309-0526, USA

**Keywords:** cell migration, cell classification, interacting particle system, equation learning, weak-form sparse identification of nonlinear dynamics

## Abstract

Interacting particle system (IPS) models have proven to be highly successful for describing the spatial movement of organisms. However, it is challenging to infer the interaction rules directly from data. In the field of equation discovery, the weak-form sparse identification of nonlinear dynamics (WSINDy) methodology has been shown to be computationally efficient for identifying the governing equations of complex systems from noisy data. Motivated by the success of IPS models to describe the spatial movement of organisms, we develop WSINDy for the second-order IPS to learn equations for communities of cells. Our approach learns the directional interaction rules for each individual cell that in aggregate govern the dynamics of a heterogeneous population of migrating cells. To sort a cell according to the active classes present in its model, we also develop a novel ad hoc classification scheme (which accounts for the fact that some cells do not have enough evidence to accurately infer a model). Aggregated models are then constructed hierarchically to simultaneously identify different species of cells present in the population and determine best-fit models for each species. We demonstrate the efficiency and proficiency of the method on several test scenarios, motivated by common cell migration experiments.

## 1. Introduction

Systems of autonomous agents are ubiquitous in the natural world. Research into their behaviour has led to a plethora of proposed mathematical models, including the agent-based ‘boids’ model [1], ordinary differential equation models for milling and flocking [2,3], and non-local partial differential equations [4,5], to name a few. A general framework for rigorous analysis of these models is by now very mature [6].

Identifying the rules of interaction between agents is necessary for predicting and influencing the cooperative abilities of any such system, whether composed of autonomous robots, large multi-cellular animals, single-celled organisms or even molecules. Methods for inferring the rules of interaction between agents using observed trajectory data have continued to advance since the early 2000s. Several of the principled techniques include force-matching [7,8], linear regression [9,10], mean-field formulations [11,12], information-theoretic tools [13], underdamped Langevin regression [14,15], Gaussian processes [16] and even a method based on topological rather than metric distances [17].

These and related techniques have been successfully used to identify the dominant drivers of collective behaviour in a variety of social and biological systems [18–20], including schools of fish [21,22], flocks of birds [23,24] and pedestrian traffic [25], all directly incorporating measured trajectory data. While popular methods, such as force-matching, are useful in identifying fields of vision and spatial statistics of interactions, they cannot easily disentangle the combined effects of multiple forces (e.g. attraction, repulsion and alignment) [26,27], let alone different interactions between multiple species of neighbours. This limits the classes of models they can identify and implies that new methods must be developed for heterogeneous populations.

The field of *equation discovery* is a highly active area of research [28–36], as it offers tools to directly learn governing differential equations. This approach is not only useful in prediction and validation, but can be used to simultaneously identify multiple active modes of inter-agent communication, such as repulsion, velocity alignment and attraction. In this work, we tackle the problem of identifying governing equations for an interacting particle system (IPS) with multiple interacting species. Our proposed approach is completely naive with regard to species membership in order to specifically address problems of heterogeneity in collective cell migration [37]. In accordance with our biological motivations, we refer to agents throughout as ‘cells’, particle systems as ‘populations’, and different cell types as ‘species’, however cell types need not correspond to ‘species’ in the biological sense (e.g. ‘leader’ and ‘follower’ cells could be classified as two different ‘species’).

Motivated by existing hypotheses regarding the anistropy of cell–cell interactions [38–40], we introduce our framework in the context of *directional interaction* models, as defined below. Moreover, we note that the documented significance of anisotropic interactions in general collective systems [41–43] suggests that our approach may have wide applicability.

### 1.1. Heterogeneous populations

Many collective populations arising in nature are inherently heterogeneous, with the rules of interaction varying across different subsets of the population. This is readily observable in complex mammalian populations, but is also seen in simpler organisms, such as honeybee swarms, where bees divide into scout and worker bee roles [44]. The advantages of heterogeneity in collective behaviour have even inspired search optimization algorithms [45,46].

At the level of microorganisms, cells have been observed to adopt leader-like and follower-like roles during collective migration events such as wound healing [47,48], without the aid of a central nervous system. Individual cell speed and persistence of motion have also been determined to be functions of the age and size of the cell [49–51], which may lead to heterogeneous responses to stimuli from neighbouring cells. The mechanisms which produce these heterogeneities, and the extent to which heterogeneity is present in a given cell population, are current subjects of debate [52–54]. Data-driven techniques may be useful in formulating accurate mathematical models in the presence of heterogeneity.

Zhong *et al*. [55] develop a highly versatile method for inferring explicit rules of interaction in a heterogeneous population, although it is assumed that species membership is known *a priori*. Several recent works have offered methods of assessing the degree of population heterogeneity [18,53], yet these methods do not provide explicit mathematical models for the different populations. By contrast, the method presented here allows one to classify the given population into different species according to the heterogeneous interaction rules present and produces explicit mathematical models for each species as a by-product.

In this work, we restrict our attention to the case where individuals within the population may follow different interaction rules, but each individual applies only one set of interaction rules to all others members of the population. In other words, individual *i* applies the same set of rules to individual *j* and *k*, while *j* and *k* may each apply different interaction rules to particle *i*. We leave the case of individual *i* interacting differently with individuals *j* and *k*, depending on the species membership of *j* and *k*, to future work.

### 1.2. Directional interaction forces

It is now well known that simple radial interaction models are incapable of explaining many observed collective behaviours in biological settings, and that directionally dependent interaction rules, based on a limited field of view or sensing angle, offer a significant advantage [23,41,56–58]. At the cellular level, directional dependence of cell–cell interaction has been proposed in the context of intracellular polarization [39]; however, the cellular sensing range is not immediately obvious, since a migrating cell does not have an obvious ‘field of view’. Recent works have sought to quantify the degree to which interactions are density-dependent [59], but not which directional modes (radial, dipolar, quadrupolar, etc.) are dominant during a collective migration event.

In addition to providing an explanation for certain observed phenomena [43], directional interaction rules are capable of generating *spontaneous migration*, due to the total directional force between particles not being conserved in general. In the modelling of active matter systems (such as migrating cells) [11,60], such symmetry breaking is commonly generated by a combination of Brownian forcing and a self-propulsion device [61]. However, it is not clear that self-propulsion is an appropriate mechanism for modelling cellular movement (in comparison with fish, which are constantly swimming). Directional forces may then be an important mechanism for symmetry breaking and spontaneous cellular migration.

### 1.3. Weak-form sparse identification of nonlinear dynamics

At its core, our method involves learning ordinary differential equations for cells using available trajectory data. For this we employ the weak-form sparse identification of nonlinear dynamics algorithm (WSINDy), which has been shown to successfully identify governing equations from data at the levels of ordinary different equations [62], partial differential equations [63], first-order interacting particle systems [12] and even works in a small-memory online streaming scenario [64].

A significant advantage of the WSINDy method is that it identifies a single governing equation which can be interpreted, analysed and simulated using conventional techniques of applied mathematics. It does not involve any black-box algorithms or mappings as would be generated in using a neural network-based approach. Another promising direction is a hybrid approach, such as [65], where the authors first learn a neural network model of the potential and then use sparse identification to learn the algebraic form of the potential. Ultimately, an interpretable sparse model provides the best chance at both describing and modelling the dynamics.

Several alternative methods have been developed to accomplish the equation learning task for particle systems. In particular, Lu *et al.* [10] develop a method for learning general feature-dependent second-order interaction rules for heterogeneous populations, where features may include directional interaction forces, speed dependence and so on. The differences between this and our work are the following. (i) We are performing the *unsupervised* learning task of classifying agents by their interaction rules, whereas Lu *et al*.’s work assumes knowledge of the species membership. (ii) We are interested in *sparse model representations*, in particular selection of the correct modes of interaction (e.g. attractive, repulsive, alignment and drag force), whereas Lu’s work assumes knowledge of both the feature-dependence and types of forces present (e.g. for planetary systems, *a priori* knowledge is used to rule out the presence of an alignment force). (iii) Lastly, models are initially extracted from *single-cell trajectories*. As described in the next section, rather than aggregating *data* which may come from multiple cell species, we aggregate *models* which are likely to describe the same species, and then use the aggregate model to perform classification.

### 1.4. Single-cell learning and model clustering

With a possibly heterogeneous population of cell trajectories available, one is tasked with the problem of deciding how to aggregate the data. If knowledge of the underlying species membership is available, a more accurate model can be inferred by pooling data from all individuals of a given species. On the other hand, pooling data from multiple species into a single model can result in a highly *inaccurate* model if very different interaction rules from multiple species are averaged together. In general, there exists a spectrum of possible pooling strategies, ranging from learning *few models* from *large subsets* of the population, to learning *many models* from *small subsets* of the populations. The former intrinsically produces models with high bias and low variance, while the latter produces models with low bias and high variance. Such pooling strategies have been recently explored in [66], where it is found that identifying a single model can be improved by pooling models learned from subsets of the data. However, this has not been extended to classifying the data itself into species, and finding a model for each species. Moreover, the IPS setting offers a particular advantage on the subject of model validation, as data can easily be assimilated into forward simulations.

In this work, we investigate the extreme case of learning an individual model $M_{i}$ for the *i*th individual trajectory, and then clustering the set of learned models $M\;:=\{M_{1},\ldots ,M_{N}\}$ according to their identified modes of interaction. This approach is counterintuitive because there is no guarantee that a single-cell trajectory will provide enough information on the interaction rules of its species. To be able to classify cells using the (potentially) insufficiently informative trajectories, we developed an ad hoc recursive classifier which we show (in §4) accurately clusters and sorts the models into species. This approach prevents any contamination that may result from combining trajectories of multiple species.

Once the models are clustered, an aggregate model $\bar{M}$ is computed by averaging the models in M belonging to the most populous cluster. The model $\bar{M}$ is then used to classify cells via forward simulations which are made highly efficient by directly incorporating the data. In particular, for each trajectory in the dataset, we use $\bar{M}$ to simulate a new trajectory, but with all neighbour interactions computed using the data. That is, only the new trajectory is propagated forward in time by model $\bar{M}$, while the rest of the population is simply the data itself. This can then be trivially parallelized, reducing an $O(N^{2})$ computational cost per time step to *N* cores performing O(N) updates per time step with no communication overhead.

We show through examples below that this hierarchical model-pooling and validation procedure produces both correct species classification and accurate governing equations, despite individual cell trajectory data carrying low levels of information. For further information on the classification algorithm, see §3.

### 1.5. Paper outline

In §2, we discuss the general form of directional interacting particle models that will be assumed in the learning process. In §3, we introduce our model selection and classification algorithm, which is composed of the six steps: (a) learn single-cell models, (b) replace inaccurate models, (c) cluster learned models according to active force modes, (d) form an aggregate model by averaging models in the largest cluster, (e) validate the aggregate model using data-driven forward simulations, and (f) classify cells according to performance under the aggregate model. In §4, we examine the performance of the algorithm in learning and classifying homogeneous and heterogeneous populations of one, two and three species. We discuss possible next directions in §5. Some additional information and a summary of notation are included in appendix A.

## 2. Directional interacting particle models

We use a general second-order directional interaction model framework, where the position and velocity $(x_{i},v_{i})\in R^{2d}$ of cell *i* in *d* spatial dimensions are governed by the differential equations

2.1 $$\begin{array}{rl} {\ddot{x}}_{i} & =\frac{1}{N_{\mathrm{tot}}}\sum_{\;j=1}^{N_{\mathrm{tot}}}f_{a-r}(|x_{i}-x_{j}|,\theta_{ij})(x_{i}-x_{j})\\ & \;+\frac{1}{N_{\mathrm{tot}}}\sum_{\;j=1}^{N_{\mathrm{tot}}}f_{\mathrm{align}}(|x_{i}-x_{j}|,\theta_{ij})(v_{i}-v_{j})\\ & \;+\frac{1}{N_{\mathrm{tot}}}\sum_{\;j=1}^{N_{\mathrm{tot}}}f_{\mathrm{drag}}(|v_{i}|,\theta_{ij})v_{i}. \end{array}$$

**Figure 1.**
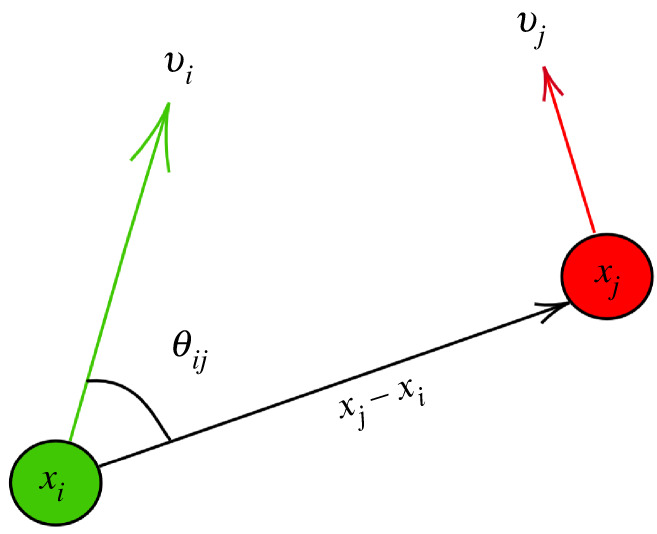
Diagram of social interactions depending on angle *θ*_*ij*_ between cell *i*’s velocity and cell *j*’s position relative to *i*.

Here, *θ*_*ij*_ is the angle between *v*_*i*_ and *x*_*j*_ − *x*_*i*_ (see the diagram in figure 1). The attractive–repulsive force *f*_a−r_, alignment force *f*_align_ and the drag force *f*_drag_ define the rules by which cell *i* communicates with the rest of the population. Our primary objective is to identify a set of interaction rules {(*f*_a−r_, *f*_align_, *f*_drag_)_ℓ_}_1≤ℓ≤*S*_, one for each of the *S* species present in the population. We note that additionally the model (2.1) can contain a stochastic noise term to capture random environmental forces; however, we leave this an extension to future work.

### 2.1. Directionality *θ*_*ij*_

As mentioned above, directional variation in the interaction forces between cells can arise from various factors, including intracellular polarization, or heterogeneous distribution of membrane-bound receptors, asymmetry in the protrusion/contraction of lamellopodia as the cell crawls on the substrate, and so on. In the current study, we assume that each of these effects is unobservable, hence we model the aggregate directional effect using the angles *θ*_*ij*_, depicted in figure 1. Dependence on angle *θ*_*ij*_ allows for interactions between cell *i* and cell *j* to vary depending on the direction of motion. Put another way, in the reference frame of cell *i*, the polar coordinates *r*_*ij*_ = |*x*_*i*_ − *x*_*j*_| and *θ*_*ij*_ allow one to represent any interaction force that varies over the two-dimensional plane.

**Figure 2.**
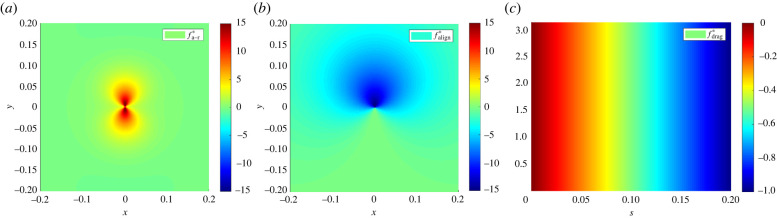
Forces used to generate artificial data, motivated by experiment. (*a*) Quadrupolar attractive-repulsive force $f_{a-r}^{⋆}$. (*b*) Dipolar alignment force $f_{\mathrm{align}}^{⋆}$. (*c*) Linear isotropic drag force $f_{\mathrm{drag}}^{⋆}$.

It should be noted that asymmetric interactions *θ*_*ij*_ ≠ *θ*_*ji*_ lead to symmetry breaking and spontaneous cell migration from an initially motionless state. In this study we restrict the angular dependence to {1, cos (*θ*_*ij*_), cos (2*θ*_*ij*_)}, which allows for a combination of radial, dipolar and quadrupolar interactions (see figure 2 for examples of dipolar (*b*) and quadrupolar (*a*) forces used in this study). Higher-order directionality can usually be assumed to be negligible; however, extension to higher modes is straightforward.

### 2.2. Attractive–repulsive force *f*_a–r_

The interaction force *f*_a−r_ acts along the vector from cell *i* to cell *j* and captures short-range repulsion and long-range signalling. Many IPS models include only an attractive–repulsive force, due to its extensive pattern-forming capabilities [67,68]. Typically *f*_a−r_ is taken to be the gradient of some potential *K*, such as the Morse potential $K(r)=C_{R}\;e^{-r/L_{R}}-C_{A}\;e^{-r/L_{A}}$ or power law potential $K(r)=r^{\;p_{R}}/p_{R}-r^{\;p_{A}}/p_{A}$, with *r* denoting the inter-particle distance. The parameters (*C*_*R*_, *L*_*R*_, *C*_*A*_, *L*_*A*_) or (*p*_*A*_, *p*_*R*_) determine the possible long-time behaviours, such as milling, spreading or concentrating.

We impose the following natural constraints on *f*_a−r_:

2.2 $$\left\{ \begin{array}{cc} f_{a-r}(r,\theta )\geq 0,\;\; & 0\leq r<r_{\mathrm{nf}}\;\;\\ f_{a-r}(r,\theta )\leq 0,\;\; & r\geq r_{\mathrm{ff}}\;\;\\ f_{a-r}(r,\theta )\in \mathrm{span}\{1,\cos(\theta ),\cos(2\theta )\}, & \text{every }r\;\text{fixed}. \end{array}\right.$$

Here *r*_nf_ is the *near-field threshold*, which can for instance correspond to a cell diameter, and *r*_ff_ is the *far-field threshold*, i.e. a large distance. The first inequality enforces that *f*_a−r_ is near-field repulsive, which must be true by volume exclusion. In practice, we define *r*_nf_ by

$$P(|x_{i}-x_{j}|<r_{\mathrm{nf}})=p_{\mathrm{nf}},$$

where in this work we set *p*_nf_ = 0.001, and the dataset is used to compute the probability, taking all interparticle distances over all time points into account. This states that the force must be repulsive over short pairwise distances which are 0.1% likely to be observed, given dataset.

The second equality enforces long-range decay, as well as model stability. Decay is natural since interactions can be expected to be small outside of some large distance *r*_ff_. We enforce that interactions are *attractive* at large distances (allowing for decay as well), so that in simulation the particles do not spread to infinity. We set *r*_ff_ = 1 throughout, although *r*_ff_ can easily be chosen from the data (e.g. *r*_ff_ = 50*r*_nf_ corresponds to an effective interaction range of 50 cell radii). (See appendix A.1 for resulting values of *r*_nf_ and *r*_ff_ and other hyperparameters for examples below.)

The third set inclusion simply reiterates the assumptions on directionality described above.

### 2.3. Alignment force *f*_align_

The alignment force *f*_align_ captures cells’ tendency to match the velocity of neighbouring cells. There are many theories as to how this arises physically [38,69]. Perhaps protrusions from cells inform the cell about the bulk direction of motion, which would be a very local effect. However, alignment models which have been proven to lead to flocking depend on sufficiently *long-range* alignment. In particular, the Cucker–Smale model involves only an alignment force, which takes the form $f_{\mathrm{align}}(r,\theta )=A/{\left(\sigma^{2}+r^{2}\right)}^{\beta }$. Unconditional flocking occurs for *β* < 1/2, and for larger *β* (leading to a shorter-range alignment force) flocking depends on the initial conditions [3].

We impose the following constraints on *f*_align_:

2.3 $$\left\{ \begin{array}{cc} f_{\mathrm{align}}(r,\theta )\leq 0,\;\; & 0\leq r\;\;\\ f_{\mathrm{align}}(r,\theta )\in \mathrm{span}\{1,\cos(\theta ),\cos(2\theta )\}, & \mathrm{every}\;r\;\mathrm{fixed}. \end{array}\right.$$

The first inequality enforces that *f*_align_ is non-positive, which is necessary for the constant velocity state *v*_*i*_ = *v*_*j*_ = *v* to be a stable configuration. If not, small perturbations away from *v*_*i*_ = *v*_*j*_ result in cells *accelerating* away from each other, which is a redundant force given that cells can be pushed away from each other through *f*_a−r_ (it is also not hard to see that *f*_align_ > 0 is unphysical). The second constraint restricts the alignment force to be a combination of radial, dipolar or quadrupolar modes, similar to *f*_a−r_.

### 2.4. Drag force *f*_drag_

The drag force *f*_drag_ captures energy expenditure due to general resistance to motion (resulting e.g. from substrate roughness); however, we allow an angular dependence on *θ*_*ij*_ to capture possible decreases or increases in drag depending on local neighbour distribution. For this we impose the following constraints:

2.4 $$\left\{ \begin{array}{cc} f_{\mathrm{drag}}(s,\theta )\leq 0,\;\; & 0\leq s<\infty \;\;\\ f_{\mathrm{drag}}(s,\theta )\in \mathrm{span}\{1,\cos(\theta )\}, & \mathrm{every}\;s\;\mathrm{fixed}, \end{array}\right.$$

where *s* indicates the speed of the cell. The force *f*_drag_ is chosen to be negative so that cells do not have a ‘self-propulsion’ device. As mentioned previously, many models of active matter include self-propulsion as a partial mechanism for symmetry breaking and general non-equilibrium effects. To reiterate, we do not expect cells to have a self-propulsion device, in fact, we wish to learn how migration occurs spontaneously, incited by pairwise interactions. In addition, a positive drag force leads to populations spreading outside of the range of meaningful interactions. In this way, negative drag is computationally beneficial, as it leads to improved model stability.

## 3. Algorithm

**Figure 3.**
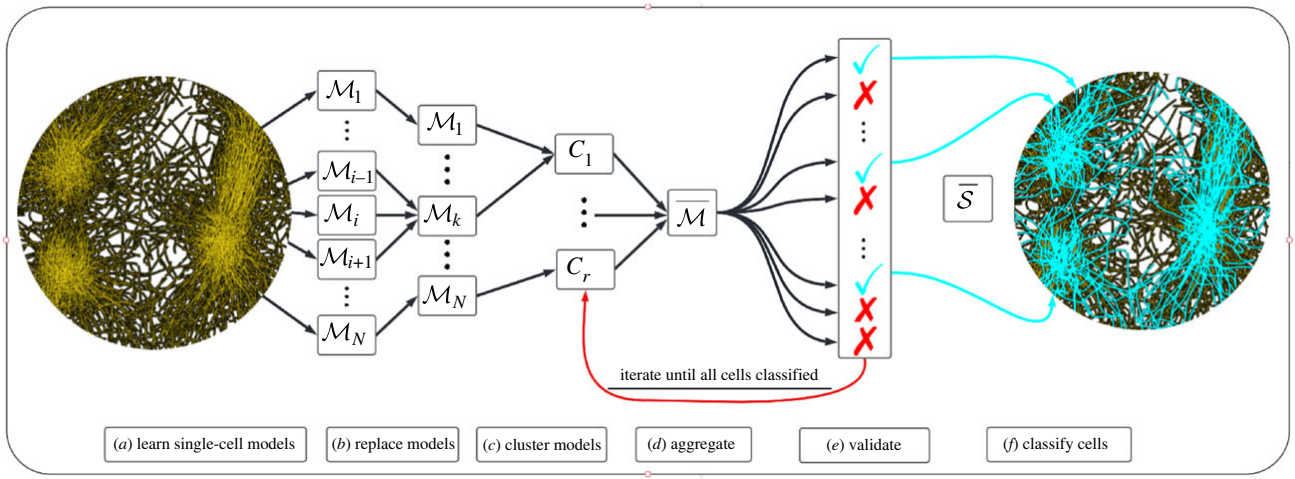
Classification pipeline for cells from heterogeneous populations. (*a*) An ensemble of models $M=\{M_{1},\ldots ,M_{N}\}$ is learned, each from an individual trajectory; (*b*) models in M are replaced by neighbouring models with superior performance if any exist; (*c*) M is partitioned into clusters $C=\{C_{1},\ldots ,C_{r}\}$ according to active forces in each model; (*d*) models in the largest cluster $\bar{C}$ are averaged together, giving $\bar{\;M}$; (*e*) $\bar{\;M}$ is validated along each individual trajectory; (*f*) validation errors are classified, producing an identified species $\bar{S}$ (cyan checkmarks) and the remaining cells (red *X*’s) are returned to step (*c*) to be clustered again. Steps (*c*–*f*) repeat until all cells are classified. Note that the number and members of model clusters C and resulting aggregate model $\bar{\;M}$ will change each iteration depending on the identity of remaining unlabelled cells.

Our algorithm involves first learning an ensemble of directional force models $M=\{M_{1},\ldots ,M_{N}\}$, that is, one model for each of the *N* *focal cells* selected for learning. Individual models $M_{i}$ are then validated on a small neighbourhood of cells, and $M_{i}$ is replaced by $M_{j}$ if a model $M_{j}$ is found that outperforms $M_{i}$ on cell *i*. We next group models into clusters $C\;:\;=\{C_{1},\ldots ,C_{r}\}$ and compute an aggregate model $\bar{\;M}$ from the largest cluster, denoted by $\bar{C}$. A new species $\bar{S}$ is then identified, with membership in $\bar{S}$ determined by the accuracy of data-driven forward simulations of model $\bar{\;M}$. Cells in the species $\bar{S}$ are then removed from the population and the remaining cells are returned to the clustering phase. More explicitly, the algorithm is composed of the following steps, which are visualized in figure 3.

(a) **Identify** individual cell models $M=\{M_{1},\ldots ,M_{N}\}$ using the WSINDy algorithm.
(b) **Replace** models in M with superior models of ‘neighbouring’ cells (as described in §3.2).
(c) **Cluster** M into $\left\{C_{1},\ldots ,C_{r}\right\}$ according to active force modes.
(d) **Aggregate** models in the largest cluster $\bar{C}$ to arrive at a single model $\bar{\;M}.$
(e) **Validate** model $\bar{\;M}$ on each remaining unlabelled cell, using the data to calculate neighbour interactions.
(f) **Classify** cells based on simulation error under $\bar{M}$ and label the lowest-error class as the new species $\bar{S}$ (remove cells in $\bar{S}$ from the population and return to step (c)).

The result is a set of *S* models and species ${\left\{({\bar{M}}_{\ell },{\bar{S}}_{\ell })\right\}}_{\ell =1}^{S}$, where each model ${\bar{M}}_{\ell }$ is constructed from an average of individual models within a cluster. We use the notation^1^ of $M_{i}$ to be the model for the *i*th cell, $C_{j}$ to be the *j*th cluster of models and ${\bar{\;S}}_{\ell }$ to be the set of cells identified as the ℓth species and which obey model ${\bar{M}}_{\ell }$.

We now give more detail on steps (a)–(f), including stopping criteria, leaving the more technical aspects to the appendix. In appendix A.1, we include a table of notations used throughout (table 5), along with algorithmic hyperparameters and their corresponding values used in the examples below, followed by a brief discussion about the problem-dependent nature of several hyperparameters.

### 3.1. Learning single-cell models

The first step in the algorithm is to learn an ensemble of *single-cell* models, one for each of the *N* focal cells selected from the *N*_tot_ total cells tracked during the experiment.^2^ By ‘single-cell’ model, we mean that the scope of each model is limited to learning only the dynamics of its focal cell; however, data from the remaining tracked cells are incorporated to learn the interaction forces.

#### 3.1.1. Weak-form sparse identification of nonlinear dynamics

The main ingredient in learning single-cell models in M is the WSINDy algorithm, together with careful choices for the bases used to represent the three main forces *f*_a−r_, *f*_align_, and *f*_drag_. Each cell *i* is identified by a position and velocity (*x*_*i*_(*t*),*v*_*i*_(*t*)) which we assume is well-approximated by a second-order model of the form (2.1). The dynamics take the general form

3.1 $${\ddot{x}}_{i}(t)=F_{i}(X(t),V(t)),$$

where $(X(t),V(t))\in R^{2dN_{\mathrm{tot}}}$ denotes the entire population of positions and velocities in the colony at time *t*. We then assume that we have available a dataset of positions $X=(x_{1}(t_{k}),\ldots ,x_{N_{\mathrm{tot}}}{\left(t_{k})\right)}_{k=1}^{L}$ sampled from the system *X* at *L* time points. Our goal is to identify *F*_*i*_ using **X**.

The SINDy approach involves representing *F*_*i*_ as a sparse linear combination of basis elements $\Theta (X,V)\;:={\left(f_{j}(X,V)\right)}_{1\leq j\leq J}$, such that

$$F_{i,d^{\prime }}(X,V)=\sum_{\;j=1}^{J}{w^{⋆}}_{i,j}f_{\;j,d^{\prime }}(X,V),$$

where subscript *d*^′^ indicates the spatial coordinate (*d*^′^ ∈ {1, 2} in this study). The basis Θ is chosen by the user and determines the accuracy of the learned model as well as the conditioning of the WSINDy algorithm.

The available cell position data **X** is used to approximate velocities $V\;:=\dot{X}\approx \dot{X}$ and accelerations $\ddot{X}\approx \ddot{X}$, using e.g. finite differences, leading from (3.1) to the data-driven linear system

3.2 $${\ddot{x}}_{i}\approx \Theta (X,V){w^{⋆}}_{i}.$$

With some abuse of notation, we denote by Θ(X,V) the matrix that results from evaluating the basis Θ(X,V) at the time-series data (**X**, **V**). The entries are $\Theta {\left(X,V\right)}_{k+(d^{\prime }-1),j}=f_{\;j,d^{\prime }}(X(t_{k}),V(t_{k}))$.

The data **X** are often corrupted by measurement noise, which leads to inaccurate computations of derivatives **V**. For the current setting, the standard SINDy approach just outlined requires *second-order* derivatives $\ddot{X}$, which are even less accurate to compute from noisy data. To prevent some of the corruption from noise,^3^ we can use the weak form, which leads to WSINDy. Returning to equation (3.1), we convert to the weak form by multiplying by a *test function* *ϕ*(*t*) and integrating in time,

3.3 $$\left\langle \phi ,{\ddot{x}}_{i}\right\rangle \;:=\left\langle \phi ,F_{i}(X,V)\right\rangle .$$

where the inner product 〈 · , · 〉 denotes the time integral

$$\left\langle f,g\right\rangle =\int_{-\infty }^{\infty }f\;(t)g(t)\;dt.$$

Choosing *ϕ* to be twice differentiable and zero outside of some interval (*a*, *b*), we then integrate by parts twice on the left-hand side to arrive at

$$\langle \ddot{\phi },x_{i}\rangle =\left\langle \phi ,F_{i}(X,V)\right\rangle ,$$

so that the second derivative has been removed from *x*_*i*_ and placed on *ϕ*. Choosing a basis of test functions $\Phi \;:={\left(\phi_{q}\right)}_{1\leq q\leq Q}$, we build the weak-form linear system

3.4 $$b^{\left(i\right)}\approx G^{\left(i\right)}{\hat{w}}^{\left(i\right)},$$

where $b_{q+(d^{\prime }-1)}^{\left(i\right)}=\langle {\ddot{\phi }}_{q},x_{i,d^{\prime }}\rangle$ and $G_{q+(d^{\prime }-1),j}^{\left(i\right)}=\left\langle \phi_{q},f_{\;j,d^{\prime }}(X,V)\right\rangle$.

As well as choosing Θ, in order to compute (**G**^(*i*)^, **b**^(*i*)^), we need to compute^4^ **V** from the position data **X**, choose a test function basis Φ and discretize integrals appearing in the linear system. For simplicity, we compute **V** using second-order centred finite difference, although a number of methods exist for numerical differentiation from data [70,71]. For integration, we use the trapezoidal rule, and we use test functions of the form

3.5 $$\phi_{q}(t)=\max{\left(1-{\left(\frac{t-t_{q}}{m\Delta t}\right)}^{2},0\right)}^{p},$$

for shape parameters *m* and *p*, and timestamps *t*_*q*_ in the range of the available time series. We use the class of test functions (3.5) for its desirable accuracy and robustness properties combined with the trapezoidal rule [62], and refer to [62,63] for methods of choosing (*m*, *p*, *t*_*q*_). In this work, we use the changepoint algorithm in [63] with *τ* = 10^−10^ and $\hat{\tau }=3$, leading to *m* ∈ {31, …, 38} and *p* ∈ {8, 9} (table 6 lists these values used in the examples below). Since the time series below are relatively short (*L* = 200 or *L* = 400), we use all available *t*_*q*_, i.e. ${\left(t_{q}\right)}_{q=1}^{Q}=(m\Delta t,\ldots ,(L-m-1)\Delta t)$ so that *Q* = *L* − 2*m*.

#### 3.1.2. Trial basis functions

In the case of the directional force model (2.1), we require three bases $F_{a-r}$, $F_{\mathrm{align}}$ and $F_{\mathrm{drag}}$ for the three proposed forces *f*_a−r_, *f*_align_ and *f*_drag_. We seek a sparse model, and so choose global basis functions, rather than a model composed of a large sum over basis functions that are spatially localized.

For the attractive–repulsive basis $F_{a-r}$ we choose products of cosines and scaled and weighted Laguerre polynomials,

3.6 $$F_{a-r}={\left\{\cos(n\theta )p_{\ell }(\alpha r)\;e^{-(\alpha /2)r}\right\}}_{n=0,\ell =0}^{2,17}$$

for ℓth degree Laguerre polynomial *p*_ℓ_. The scale *α* is chosen from *r*_max_, the maximum observed distance between cells, such that $e^{-(\alpha /2)r_{\max}}=\epsilon_{\mathrm{mach}}\approx e^{-36}$. We set *α* = 36 in all cases below since *r*_max_ ≈ 2.

The pattern of attractive and repulsive regions of the force *f*_a−r_ is not known *a priori*, hence the Laguerre basis offers flexibility. The choice of weighted Laguerre polynomials (with weight *ω*(*r*) = e^−*r*/2^) is guided by the orthogonality relation

3.7 $$\int_{0}^{\infty }p_{m}(r)p_{n}(r)\omega^{2}(r)\;dr=\delta_{mn},$$

where *δ*_*mn*_ is the Kronecker delta. We find $F_{a-r}$ leads to a well-conditioned matrix **G** despite orthogonality not holding with respect to the data distribution. We use the first 18 such weighted Laguerre polynomials to provide a sufficiently large basis; however, this number is fairly arbitrary and may need to be increased or decreased depending on the complexity of the dynamics.

For the alignment force, we choose a basis of shifted cosines and exponential functions

3.8 $$F_{\mathrm{align}}={\left\{(1+\cos(n\theta ))\;e^{-2^{\ell }r}\right\}}_{n=0,\ell =-2}^{2,5}$$

which is informed by the fact that *f*_align_ must be negative. This is easily controlled with $F_{\mathrm{align}}$ by simply enforcing that the coefficients ${\hat{w}}_{\mathrm{align}}$ be negative. For the same reason, we choose the drag force from a basis of monomials and cosines,

3.9 $$F_{\mathrm{drag}}={\left\{(1+\cos(n\theta ))|v{|}^{\ell }\right\}}_{n=0,\ell =0}^{1,4}$$

as this can also be easily controlled to yield an overall negative *f*_drag_ force by constraining only the basis elements ${\hat{w}}_{\mathrm{drag}}$. Moreover, monomials capture the physical assumption that resistance to motion should increase with speed.

#### 3.1.3. Regression

We solve the linear system (3.4) for coefficients ${\hat{w}}^{\left(i\right)}$ by approximately solving the following constrained sparse regression problem:

3.10 $${\hat{w}}^{\left(i\right)}=\underset{w\;s.t.\;Cw\leq d}{\arg\;\min}\{\|G^{\left(i\right)}w-b^{\left(i\right)}{\|}_{2}^{2}+\lambda^{2}\|w{\|}_{0}\}.$$

The linear inequality constraint **Cw** ≤ **d** encodes the constraints listed in (2.2), (2.3) and (2.4) on the forces on *f*_a−r_, *f*_align_ and *f*_drag_, and *λ* is the sparsity threshold. We employ the modified sequential thresholding algorithm from [12,63], with least-squares iterations replaced by solving the associated linearly constrained quadratic program.^5^

Since the coefficients ${\hat{w}}^{\left(i\right)}$ have no *a priori* absolute magnitude, we threshold only on the magnitudes of the given term relative to the response vector **b**^(*i*)^, namely, we define the thresholding operator $H_{\lambda }(w)$ by

3.11 $${\left(H_{\lambda }(w)\right)}_{j}=\{\begin{array}{cc} 0,\; & \frac{\|G_{j}^{\left(i\right)}{\left(w\right)}_{j}\|}{\|b^{\left(i\right)}\|}\notin [\lambda ,\;\;\lambda^{-1}]\;\;\\ {\left(w\right)}_{j},\; & \mathrm{otherwise}.\; \end{array}$$

The sequential thresholding algorithm for solving (3.10) thus produces iterates $\left\{w_{0}^{\left(i\right)},\;\ldots ,w_{\ell }^{\left(i\right)},\;\ldots ,{\hat{w}}^{\left(i\right)}\right\}$ where each $w_{\ell +1}^{\left(i\right)}$ is obtained from $w_{\ell }^{\left(i\right)}$ by first solving (3.10) with *λ* = 0 for $\tilde{w}$ subject to $\mathrm{supp}(\tilde{w})\subset \mathrm{supp}(w_{\ell }^{\left(i\right)})$, and then setting $w_{\ell +1}^{\left(i\right)}=H_{\lambda }(\tilde{w})$. A sweep over 40 equally log-spaced *λ* values $\lambda =(10^{-4},\ldots ,1)$ is performed according to [12,62] to choose an appropriate threshold *λ*.

### 3.2. Model replacement

Model replacement is akin to ‘cross-pollination’ and is crucial to increasing the accuracy of the learned models, as it transfers successful learning of few cells with highly informative trajectories to cells with less informative trajectories. As with all validation steps of our algorithm, this approach would be infeasible if not for fast data-driven forward simulations, as discussed further in §3.5.

Once the initial batch of *N* models M is learned, we simulate each model $M_{j}$ as outlined in §3.5 on *K* different *validation cells* selected from the data and specific to cell *j*, where we set *K* = 32 throughout. If $M_{j}$ performs better than $M_{i}$ on cell *i*, we replace $M_{i}$ with $M_{j}$ (specifically, $M_{i}$ is replaced with the *best performing* such model, if one exists).

For a given model $M_{i}$, we select the *K* validation cells by finding cells in the population that match well certain statistics of cell *i*. In particular, we define the following distributions:

3.12 $$\rho_{rr}^{\left(i\right)}(r)=\frac{1}{T}\int_{0}^{T}P_{x∼X^{\prime }}(\|x_{i}(t)-x(t)\|<r)\;dt,$$

3.13 $$\rho_{vv}^{\left(i\right)}(s)=\frac{1}{T}\int_{0}^{T}P_{v∼V^{\prime }}(\|v_{i}(t)-v(t)\|<s)\;dt$$

3.14 $$\mathrm{and}\;\;\;\rho_{v}^{\left(i\right)}(s)=P(\|v_{i}\|<s),$$

where (*X*^′^, *V*^′^) denotes the remainder of the cell population excluding cell *i*. Respectively, these denote the distribution of *distances* from cell *i* to all other cells, the distribution of *velocity differences* between cell *i* and all other cells, and the distribution of *speeds* that cell *i* experiences. These statistics are likely to correspond to the information content that cell *i* carries about its own forces *f*_a−r_, *f*_align_ and *f*_drag_, given the force dependencies. We approximate these distributions from the data using histograms with 50 bins. Figures 15 and 16 in the appendix depict species averages of $\rho_{rr}^{\left(i\right)}$, $\rho_{vv}^{\left(i\right)}$, $\rho_{v}^{\left(i\right)}$.

For each cell *i*, we compute the Kullback–Leibler (KL) divergence between its distributions $\rho_{rr}^{\left(i\right)}$, $\rho_{vv}^{\left(i\right)}$, $\rho_{v}^{\left(i\right)}$ and those of the rest of the population,^6^ where the KL divergence between densities *ρ* and *ν* is given by

$$D_{\mathrm{KL}}(\rho |\nu )=-\int \rho (x)\log\left(\frac{\nu (x)}{\rho (x)}\right)\;dx.$$

The *K* validation cells used to validate model *i* are the *K* cells with smallest cost L, defined by

$$L\;:=D_{\mathrm{KL}}{\left(\rho_{rr}^{\left(i\right)}|\rho_{rr}^{\left(j\right)}\right)}^{2}+D_{\mathrm{KL}}{\left(\rho_{vv}^{\left(i\right)}|\rho_{vv}^{\left(j\right)}\right)}^{2}+D_{\mathrm{KL}}{\left(\rho_{v}^{\left(i\right)}|\rho_{v}^{\left(j\right)}\right)}^{2}.$$

Let the validation error Δ*V*_*i*→*j*_ be defined as in (3.19), but indicating $M_{i}$ used to validate cell *j* (i.e. using the initial conditions of cell *j*). We replace $M_{i}$ with $M_{j}$ if the following three conditions are met:

(1) Δ*V*_*i*→*i*_ > Δ*V*_*j*→*i*_
(2) Δ*V*_*i*→*j*_ > Δ*V*_*j*→*j*_
(3) max{Δ*V*_*j*→*i*_, Δ*V*_*j*→*j*_} < tol,

where we set tol = 0.25 in this work. In words, $M_{j}$ replaces $M_{i}$ if (1) $M_{j}$ performs better than $M_{i}$ on cell *i*, (2) $M_{j}$ performs better than $M_{i}$ on cell *j*, and (3) $M_{j}$ achieves a reasonably low error (defined by tol) on both cell *i* and cell *j*. (Note that cell *i* and cell *j* are required to be mutual validation cells for a model replacement to occur). Furthermore, if $M_{j}$ replaces $M_{i}$, and another model $M_{k}$ replaces $M_{j}$, we replace $M_{i}$ with $M_{k}$ as well, even if cells *i* and *k* are not mutual validation cells.

### 3.3. Cluster

Models are then clustered according to the force modes present. Specifically, using the bases above, we can expand each force according to distinct directional modes

$$\begin{array}{rl} & f_{a-r}(r,\theta )=f_{a-r}^{\left(0\right)}(r)+\cos(\theta )f_{a-r}^{\left(1\right)}(r)+\cos(2\theta )f_{a-r}^{\left(2\right)}(r),\\ & f_{\mathrm{align}}(r,\theta )=f_{\mathrm{align}}^{\left(0\right)}(r)+\cos(\theta )f_{\mathrm{align}}^{\left(1\right)}(r)+\cos(2\theta )f_{\mathrm{align}}^{\left(2\right)}(r)\\ \mathrm{and}\; & f_{\mathrm{drag}}(|v|,\theta )=f_{\mathrm{drag}}^{\left(0\right)}(|v|)+\cos(\theta )f_{\mathrm{drag}}^{\left(1\right)}(|v|). \end{array}\;\;$$

This leads to eight possible force modes, which we order as follows:

3.15 $$\{f_{a-r}^{\left(0\right)},f_{a-r}^{\left(1\right)},f_{a-r}^{\left(2\right)},f_{\mathrm{align}}^{\left(0\right)},f_{\mathrm{align}}^{\left(1\right)},f_{\mathrm{align}}^{\left(2\right)},f_{\mathrm{drag}}^{\left(0\right)},f_{\mathrm{drag}}^{\left(1\right)}\}.$$

We associate the sparsity pattern of the force modes with the set of all 8-bit codes, giving a total of 2^8^ = 256 possible model clusters. Models are partitioned into clusters $C=\{C_{1},\;\ldots ,C_{r}\}$ based on their associated codes. For example, species *A* listed in table 1 is associated with the code 10111010 indicating that $f_{a-r}^{\left(0\right)}$, $f_{a-r}^{\left(2\right)}$, $f_{\mathrm{align}}^{\left(0\right)}$, $f_{\mathrm{align}}^{\left(1\right)}$ and $f_{\mathrm{drag}}^{\left(0\right)}$ are present in the model.

**Table 1.** Species delineation by active force modes.

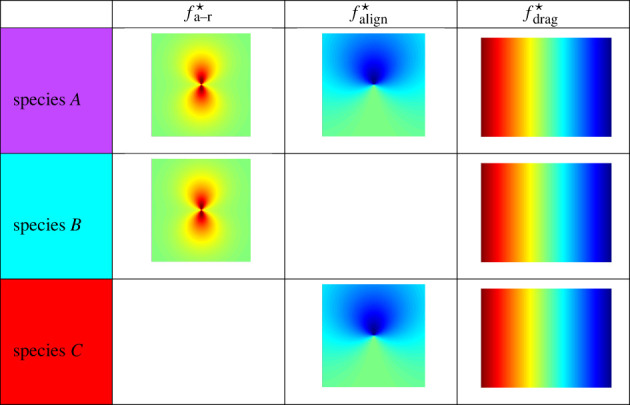

There are several other options for model replacement and clustering, include clustering based on the sparsity pattern of $\hat{w}$, or simply on the presence or the absence of each of the three forces *f*_a−r_, *f*_align_ and *f*_drag_. The former significantly increases the number of possible clusters, while the latter leads to just 8 possible clusters. Our choice reflects the desire to disentangle directionalities of the governing forces without introducing a strong dependence on the bases used to approximate each force.

### 3.4. Aggregate

Having formed the model clusters C, let $\bar{C}$ be the cluster with the most members. We then compute $\bar{M}$ by averaging^7^ over the coefficients from models in $\bar{C}$ and then performing a final round of thresholding. That is, we compute

3.16 $$\begin{array}{cc} & \bar{w}=\frac{1}{\left|\bar{C}\right|}\sum_{i\in \bar{C}}{\hat{w}}^{\left(i\right)},\;\;\\ & I=\{i\;:\;|{\bar{w}}_{i}|<10^{-\lambda_{\log}}\max|\bar{w}|\}\\ \mathrm{and}\; & \bar{w}(I)=0,\;\; \end{array}\}$$

where $\left|\bar{C}\right|$ denotes the number of elements in $\bar{C}$ and *λ*_log_ = 4 in this work, so that coefficients falling below four orders of magnitude from the maximum absolute coefficient are discarded. Thresholding here is simply to speed up computation, as small coefficients result in unnecessary evaluation of negligible basis functions during forward solves.

### 3.5. Validate

To validate the aggregate model $\bar{M}$, we perform forward simulations over the remaining unclassified cells in a highly parallelizable way that uses the experimental data to efficiently march forward in time.

Let *N*′ ≤ *N* be the number of remaining unclassified cells. For each *i* = 1, …, *N*′, we simulate a new trajectory ${\left\{({\bar{x}}_{i}(t_{k}),{\bar{v}}_{i}(t_{k}))\right\}}_{k=1}^{L}$ using the averaged model $\bar{\;M}$ with the experimental initial conditions $\left({\bar{x}}_{i}(0),{\bar{v}}_{i}(0))=(x_{i}(0),v_{i}(0)\right)$. We march forward in time according to the forward Euler update

3.17 $${\bar{v}}_{i}(t_{k+1})={\bar{v}}_{i}(t_{k})+\Delta t\bar{M}({\bar{x}}_{i}(t_{k}),{\bar{v}}_{i}(t_{k}),X^{\prime i}(t_{k}),V^{\prime i}(t_{k}))$$

and

3.18 $${\bar{x}}_{i}(t_{k+1})={\bar{x}}_{i}(t_{k})+\Delta t{\bar{v}}_{i}(t_{k}),$$

where (**X**^′*i*^(*t*_*k*_), **V**^′*i*^(*t*_*k*_)) indicates (**X**(*t*_*k*_), **V**(*t*_*k*_)) with the *i*th cell removed.^8^ Since the time resolution of the data Δ*t* is assumed to be coarse, we perform the simulation on a finer grid with time step Δ*t*′ = 2^−5^Δ*t*, and use piecewise cubic hermite interpolation to generate positions and velocities of neighbours (**X**^′*i*^, **V**^′*i*^) at intermediate times. We stress that we do not update the neighbour cells using the model $\bar{\;M}$, which would be much more costly; we merely use neighbour positions and velocities from the data to compute interactions that govern the motion of cell *i*. The resulting trajectories ${\left\{({\bar{x}}_{i},{\bar{v}}_{i})\right\}}_{i=1}^{N^{\prime }}$ can then be computed in a trivially parallel manner.^9^

We then define the validation error for cells *i* = 1, …, *N*′ as the relative velocity difference

3.19 $$\Delta V_{i}\;:=\sqrt{\frac{\sum_{k=1}^{L^{\prime }}\|{\bar{v}}_{i}(t_{k})\;-\;v_{i}(t_{k}){\|}_{2}^{2}}{\sum_{k=1}^{L^{\prime }}\|v_{i}(t_{k}){\|}_{2}^{2}}},$$

where *L*′ ≤ *L* is a subset of the time series over which the simulation is expected to remain accurate. In particular, for chaotic systems the trajectories cannot be expected to remain close for large times; however, the correct model will be initially accurate. In this work, we choose *L*′ = 0.25*L*. In other words, with *L* = 200 time steps (as in most examples below), we compare with the data over the first 50 time steps at the original scale Δ*t*, or equivalently 1600 time steps on the finer scale Δ*t*′.

### 3.6. Classify

**Figure 4.**
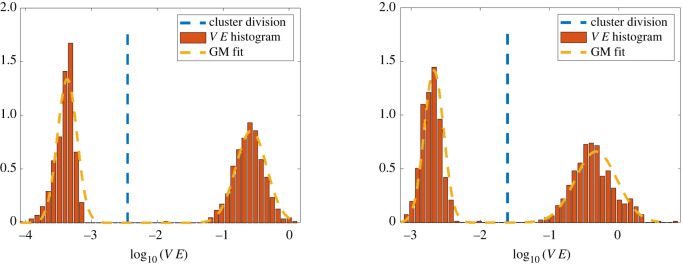
Distribution of log-validation errors for heterogeneous cell experiments **X**_*A*,*C*_ and **X**_*B*,*C*_. In each case, species *C* is identified in the first iteration, and a clear separation between the two species allows for accurate clustering using Gaussian mixture models.

**Figure 5.**
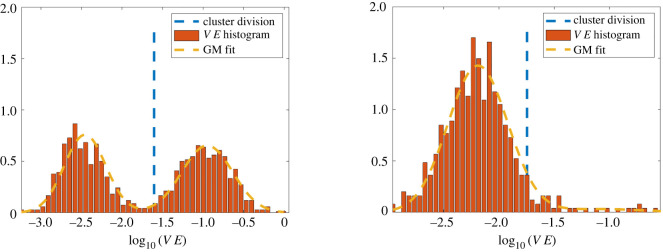
Log-validation errors for two-species population **X**_*A*,*B*_ (long). Strong similarities between the two species present an initial challenge to identification, which is overcome by taking a longer time series. The initial Gaussian mixture model (left) identifies a majority species *B* cluster. In the second iteration (right), a cluster with all species *A* cells is identified, and a small group of cells remains which is then partitioned correctly (see row 5, columns CS(*A*) and CS(*B*) of table 3).

**Figure 6.**
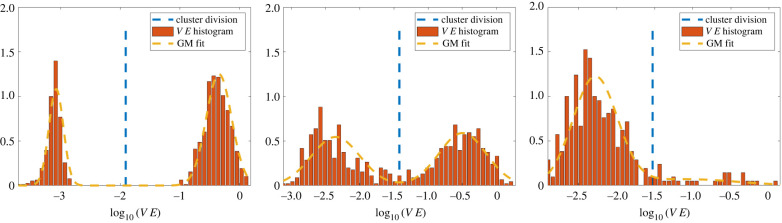
Gaussian mixture models for classifying the three-species experiment **X**_*A*,*B*,*C*_(long) (see table 4 row 3 for details). We see an initial complete separation of species *C* (left), followed by a mixed cluster containing 96.1% of the species *B* cells and 0.9% of the species *A* cells (middle). The next iteration classifies an entirely species *A* cluster (right). Clusters 4 and 5 are effectively outliers and contain the remaining 31/1000 cells.

Let VE be the set of validation errors, VE = {Δ*V*_1_, …, Δ*V*_*N*′_}. An empirical observation used in this work is that when $\bar{\;M}$ approximates well an underlying model for a true species, the log-transformed validation errors log_10_(VE) are fit well by a Gaussian mixture model (GMM) with two mixtures (see figures 4–6). We thus use a two-mixture GMM to classify the remaining cells. Cells are granted membership into the mixture that yields the highest posterior probability of generating its log-validation error, and the class with lowest mean error is labelled as a species. This can be thought of as a sequential binary classification scheme.

For example, in each plot of figure 4, a representative GMM resulting from a two-species population, the left-most mixture corresponds to low validation errors under the model $\bar{M}$ and is classified as a species $\bar{S}$ (in this case, species *C* in table 1 is identified). The cells in $\bar{S}$ are subsequently removed from the population, and cells in the right-most mixture are returned to the cluster phase (c).

### 3.7. Stopping conditions

Steps (c)–(f) are repeated until one the following conditions is met.

(1) Less than *N*′_min_ cells remain.
(2) More than $(1-\delta_{\mathrm{gmm}})\times 100\text{\%}$ of remaining cells have less than $\epsilon_{\mathrm{gmm}}\times 100\text{\%}$ validation error: $P(\mathrm{VE}<\epsilon_{\mathrm{gmm}})\geq (1-\delta_{\mathrm{gmm}})$.
(3) The maximum allowable number of species has been reached: *S* = *S*_max_.

**Figure 7.**
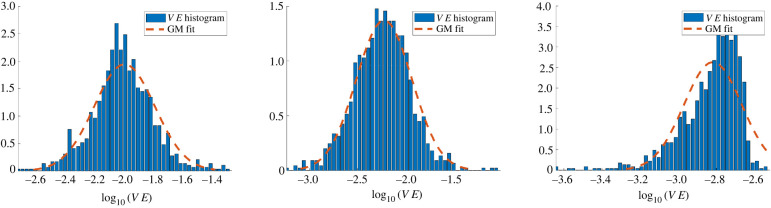
Distribution of log-validation errors for homogeneous cell experiments **X**_*A*_, **X**_*B*_, **X**_*C*_. Distributions for **X**_*A*_ and **X**_*B*_ are fit well by a single Gaussian, indicating a single species is present. The distribution for **X**_*C*_ has a non-Gaussian tail, although all errors are below 1%, indicating that the candidate model fits the population up to the specified error tolerance.

The first case is an obvious criterion to prevent infinite looping over outlier cells for which there is not enough information to learn an adequate model. We set *N*′_min_ = 2. The second condition skips the GMM fitting process when all cells have sufficiently low error. If the condition is met, all cells with error less than *ε*_gmm_ are assigned to a new species, while the remaining cells are left as outliers without a model. We choose (*ε*_gmm_, *δ*_gmm_) = (0.05, 0.01), such that if 99% of the cells achieve less than 5% error, the algorithm terminates. This is necessary to account for the case of *high-accuracy* recovery, where it is observed that *VE* is no longer approximately lognormal, leading to an inaccurate GMM partition (see e.g. the rightmost plot of figure 7). Finally, for *N* very large, it may be necessary to restrict the total number of species, which is encapsulated in the third condition. We set *S*_max_ = 10 throughout, although we did not observe the number of iterations exceeding 5 in any trials with *N* ≤ 1000 and *S* ≤ 3 true species.

## 4. Results: artificial cells

We examine artificial cell cultures with combinations of 1–3 distinct cell types, denoted by species *A*, species *B* and species *C*. Each species has a unique combination of the following forces:

4.1 $$f_{a-r}^{⋆}(r,\theta )\;:=(15+10\cos(2\theta ))(e^{-20r}-0.25\;e^{-10r}),$$

4.2 $$f_{\mathrm{align}}^{⋆}(r,\theta )\;:=-(8+8\cos(\theta ))\;e^{-8r}$$

4.3 $$\mathrm{and}\;\;\;f_{\mathrm{drag}}^{⋆}(s,\theta )\;:=-5s.$$

The forces $f_{a-r}^{⋆}$ and $f_{\mathrm{align}}^{⋆}$ are depicted in figure 2, and force combinations for species *A*, *B* and *C* are specified in table 1. The forces include a *quadrupolar* attractive–repulsive force $f_{a-r}^{⋆}$, a *dipolar* alignment force $f_{\mathrm{align}}^{⋆}$, and a *monopolar* drag force $f_{\mathrm{drag}}^{⋆}$ which is linear in its speed argument. As we will see below, species *A* and species *B* share the force *f*_a−r_ and hence result in similar dynamics, which presents a challenge to identification. It turns out that using a longer time series results in correct classification.

We let **X**_*P*_ denote a simulation with individuals from species *P*, for example, **X**_*A*_ is a simulation with only individuals from species *A* and **X**_*A*,*B*_ is a simulation with a mixed population of species *A* and species *B*. Each simulation has 1000 individuals and the same number of members in each species (up to rounding). More details on the simulations, including plots of initial and final states and several statistics, can be found in appendix A.2.

We refer to species identified by the algorithm as ‘clusters’ to disambiguate between the true species (*A*, *B*, *C*) present in the data. We are particularly interested in three traits of our learning algorithm: (1) was the classification successful? (2) are the learned forces close to the true forces? (3) are simulated trajectories using the learned model close to the original trajectories? To assess (1) we report the *classification success* CS(*i*) for *i* ∈ {*A*, *B*, *C*} as the fraction of individuals from species *i* that ended up in the cluster in question, where clusters are listed as subrows (rows not separated by horizontal lines) within each row in tables 2–4, in the order they were identified. For example, in row 2 of table 3, two clusters are identified from the two-species data **X**_*A*,*C*_, with the first cluster containing 100% of the species *C* cells, indicated by CS(*C*) = 1.000, and the second cluster containing 100% of the species *A* cells, indicated by CS(*A*) = 1.000, with no outliers.

**Table 2.** Performance of model learning and classification algorithm of homogeneous populations.

experiment	Δ*f*_a−r_	Δ*f*_align_	Δ*f*_drag_	CS(*A*)	CS(*B*)	CS(*C*)	Δ*V*
**X**_*A*_	0.0211	0.0384	0.0382	1.000	—	—	0.0100
**X**_*B*_	0.0112	—	0.0125	—	0.997	—	0.0076
**X**_*C*_	—	0.0007	0.0016	—	—	1.000	0.0016

**Table 3.** Performance of model learning and classification algorithm for two-species populations. **X**_*A*,*B*_(long) is simply the continuation of **X**_*A*,*B*_ to twice the time horizon, and significantly improves classification over **X**_*A*,*B*_. Note that identified species are listed within each delinearted row as subrows (rows not separated by horizontal lines) in the order they were identified.

experiment	Δ*f*_a−r_	Δ*f*_align_	Δ*f*_drag_	CS(*A*)	CS(*B*)	CS(*C*)	Δ*V*
**X**_*A*,*C*_	—	0.0011	0.0002	0	—	1.000	0.0005
	0.0226	0.0941	0.0472	1.000	—	0	0.0200
**X**_*B*,*C*_	—	0.0077	0.0051	—	0	1.000	0.0023
	0.0328	—	0.0461	—	1.000	0	0.0339
**X**_*A*,*B*_	0.0341	—	0.0133	0.102	0.628	—	0.1201
	0.0075	—	0.0538	0.084	0.340	—	0.0362
	0.4780	0.3660	—	–0.814	0.012	—	0.3945
**X**_*A*,*B*_(long)	0.0018	—	0.0042	0.002	0.978	—	0.0045
	0.0034	0.0023	0.0067	0.994	0	—	0.0070
	0.0071	—	0.0044	0	0.023	—	0.0568
	0.0034	0.0051	0.0144	0.004	0	—	0.0199

**Table 4.** Performance of model learning and classification algorithm of three-species populations. **X**_*A*,*B*,*C*_(long) is simply the continuation of **X**_*A*,*B*,*C*_ to twice the time horizon.

experiment	Δ*f*_a−r_	Δ*f*_align_	Δ*f*_drag_	CS(*A*)	CS(*B*)	CS(*C*)	Δ*V*
**X**_*A*,*B*,*C*_	—	0.0005	0.0020	0	0	1.000	0.0016
	0.0287	—	0.0191	0.091	0.988	0	0.0620
	0.0243	0.2321	—	0.542	0.006	0	0.5010
	0.0022	0.0001	0.0050	0.332	0	0	0.0406
	0.0478	—	0.118	0.009	0.006	0	0.3462
**X**_*A*,*B*,*C*_(long)	—	0.0014	0.0013	0	0	1.000	0.0009
	0.0082	—	0.0008	0.009	0.961	0	0.0064
	0.0041	0.0014	0.0010	0.937	0	0	0.0067
	0.0065	0.0077	0.0074	0.045	0	0	0.0482
	0.0151	—	0.0054	0.009	0.039	0	0.347

To assess the accuracy of learned forces with respect to the ground truth forces, for each of the three forces we compute the relative *L*^2^ error over a square grid discretized with 1000 points in each direction. We denote these quantities by Δ*f*_a−r_, Δ*f*_align_ and Δ*f*_drag_ in tables 2–4. For *f*_a−r_ and *f*_align_ we use (*x*, *y*) = (*r*cos*θ*, *r*sin*θ*) ∈ [ − 2, 2] × [ − 2, 2], since *r*_max_ ≈ 2 for all examples, and for *f*_drag_ we use (*s*, *θ*) ∈ [0, *s*_max_] × [0, *π*], where *s*_max_ is the maximum speed attained during the experiment. It is worth mentioning that for *f*_a−r_, we use a force that *does not* have a sparse representation in the basis $F_{a-r}$. In this case, we see that the algorithm correctly classifies individuals despite having the truncation error that results from representing the force over the basis $F_{a-r}$.

Lastly, we assess the difference in learned and true trajectories using the average validation error $\Delta V=(1/\left|\bar{S}\right|)\sum_{i\in \bar{S}}\Delta V_{i}$, where Δ*V*_*i*_ is computed from model $\bar{M}$ associated with identified species $\bar{S}$ using (3.19).

### 4.1. Homogeneous populations

**Figure 8.**
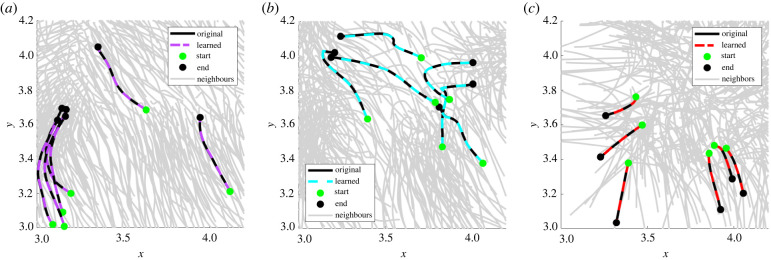
Examples of learned and original trajectories from homogeneous populations. (*a*) **X**_*A*_, (*b*) **X**_*B*_, (*c*) **X**_*C*_.

As an initial benchmark, we detect single-species populations from homogeneous data. While simpler than the heterogeneous case, this is a non-trivial task due to the variability of single-cell trajectories and local environments within the population. Our method successfully identifies the models for species *A*, *B*, and *C* from homogeneous simulations, achieving less than 1% mean validation errors in each case, and less than 4% relative force errors Δ*f* (table 2). In simulation **X**_*B*_, three cells are identified as outliers (appearing in the right tail of figure 7 (middle)), and all other cells in **X**_*A*_, **X**_*B*_, and **X**_*C*_ are correctly classified. A comparison between original and learned trajectories is depicted in figure 8, with learned trajectories overlapping original trajectories in each case.

### 4.2. Two-species populations

**Figure 9.**
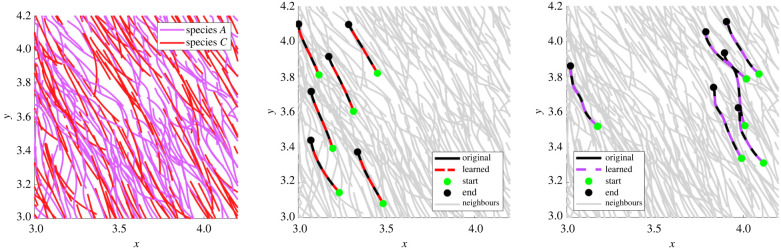
Example trajectories from experiment **X**_*A*,*C*_. Cells with true colour labels are depicted on the left, but are passed into the algorithm unlabelled. The algorithm then classifies the population into different species and returns accurate models for each species. Classified cells from species *C* (middle) and species *A* (right) are highlighted showing excellent agreement between data and simulation.

**Figure 10.**
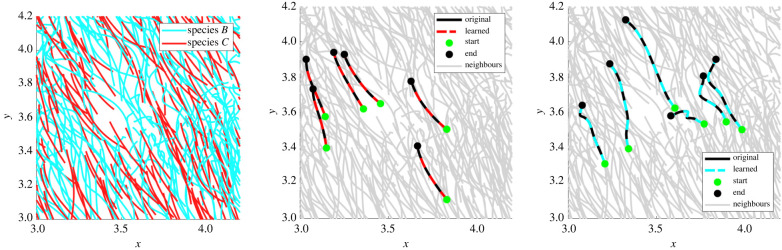
Example trajectories from experiment **X**_*B*,*C*_. As in figure 9, cells with true colour labels are depicted on the left. Classified cells from species *C* (middle) and species *B* (right) are highlighted showing excellent agreement original data and output of the learned models.

Next we examine the ability of the learning algorithm to detect two-species populations along with accurate aggregate models. Figure 4 displays two representative Gaussian mixture fits to the log-validation errors for **X**_*A*,*C*_ (left) and **X**_*B*,*C*_ (right). In both cases, the log-errors are well approximated by Gaussian mixtures with wide separations between mixtures. This allows for complete classification in both cases, as indicated by CS(*A*), CS(*B*), and CS(*C*) in rows 2 and 3 of table 3. Force differences Δ*f* are less than 5% in all but one case (estimation of *f*_align_ in cluster 2 of experiment **X**_*A,C*_), with trajectory validation errors less than 3.5%. In particular, species *C* achieves less that 0.3% validation error, which is due to the true force $f_{\mathrm{align}}^{⋆}$ existing in the span of the library $F_{\mathrm{align}}$, whereas $f_{a-r}^{⋆}$ is approximated using a truncated series expansion, resulting in larger errors. See figures 9 and 10 for comparison between original and learned trajectories.

For experiment **X**_*A*,*B*_, initially the method is incapable of correctly classifying cells into species *A* and species *B*. Three clusters are identified with suboptimal models (table 3 row 4). Accurate classification is achieved by running the algorithm with a longer experiment **X**_*A*,*B*_(long) (table 3 row 5) which is the continuation of **X**_*A*,*B*_ for twice the total time points, at the same temporal resolution. An initial cluster is identified containing 97.8% of species *B* along with 0.2% (a single cell) of the existing species *A* cells, followed by a second cluster with 99.4% of the species *A* cells and no cells from species *B*. The last two clusters correctly partition the remaining cells (12 in total), again finding accurate models, allowing for recombination with the first two cluster during post-processing.

**Figure 11.**
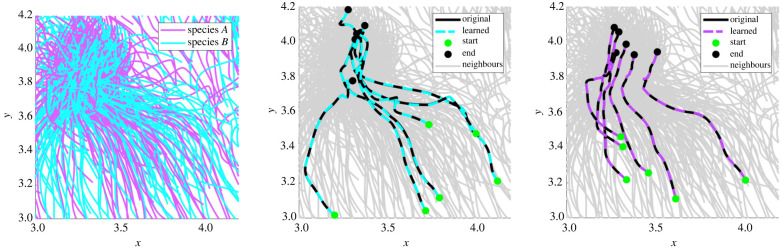
Example trajectories from experiment **X**_*A*,*B*_(long). Cells with true labels are depicted on the left and classified cells from species *B* (middle) are species *A* (right) are depicted with model output overlapping the input data in each case.

Figure 11 shows a comparison between original and learned trajectories for **X**_*A*,*B*_(long) and figure 5 depicts representative Gaussian mixture models. In particular, figure 5 (left) shows increased overlap between the two Gaussian mixtures in the first iteration, compared with figure 4; however, model performance is still sufficiently different as to classify approximately 98% of cells correctly.

### 4.3. Three-species population

As a final test we identify species from the three-species experiment **X**_*A*,*B*,*C*_. Similar to the case **X**_*A*,*B*_, we see improvements with a longer time-series **X**_*A*,*B*,*C*_(long). For the initial experiment **X**_*A*,*B*,*C*_, species *C* is completely identified in the first cluster (table 4 row 2), and in the second cluster 98.8% of species *B* cells are identified along with 9.1% of species *A* cells, leading to a fairly inaccurate model (Δ*V* ≈ 0.06). The subsequent clusters divide the remaining species *A* and *B* cells.

Doubling the time series with **X**_*A*,*B*,*C*_(long), we find the majority of each species residing in its own cluster (table 4 row 3). Cluster 1 contains all of the species *C* cells, cluster 2 consists of 96.1% of species *B* cells and 0.9% of species *A*, and cluster 3 consists of 93.7% of species *A*. Moreover, the aggregate models for each of these first three clusters result in validation errors under 1%.

**Figure 12.**
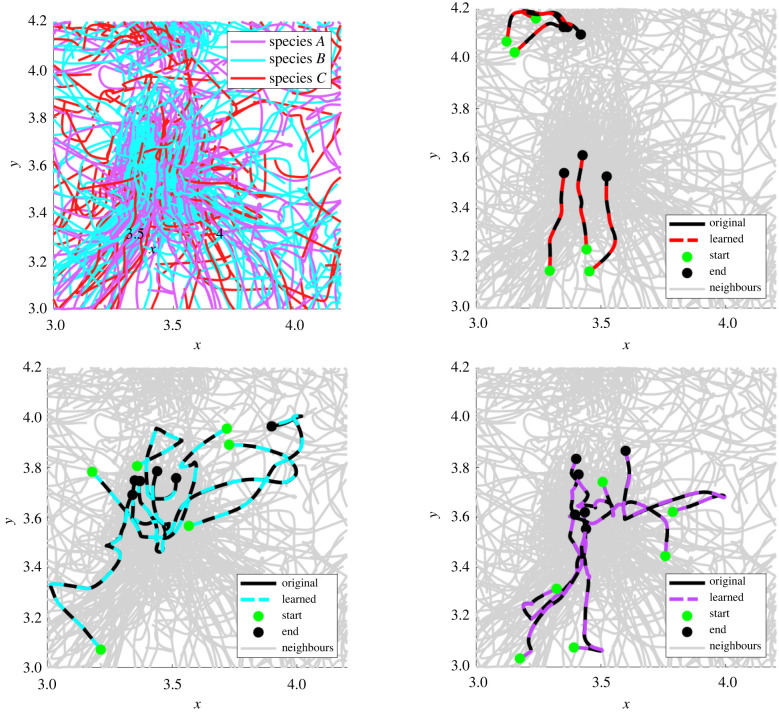
Example trajectories simulated using learned models from **X**_*A*,*B*,*C*_(long). Top left: example cells with true labels. Top right, bottom left, bottom right: example trajectories from clusters 1–3 (see table 4 row 3 for details). Note in particular that learned models for clusters 2 and 3 are able to capture sharp turns in the true dynamics.

**Figure 15.**
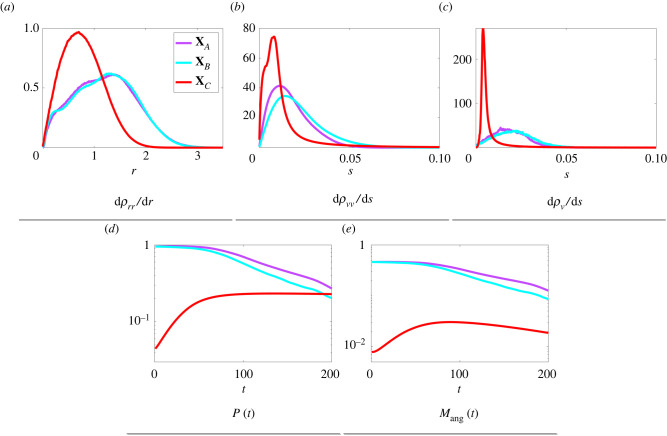
Statistical information for members of species *A*, *B* and *C* within the homogeneous experiments **X**_*A*_, **X**_*B*_, **X**_*C*_. Top row: pairwise distance density, pairwise velocity density and speed density (averages of distributions in (3.12)–(3.14) used for model replacement). Bottom row: average polarization and angular momentum over time (equation (A1)).

**Figure 16.**
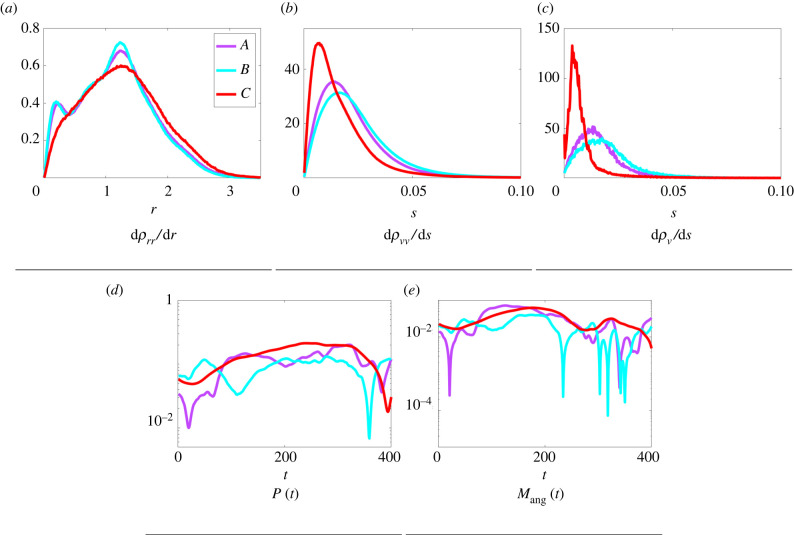
Similar to figure 15, only for members of species *A*, *B* and *C* within the single experiment **X**_*A*,*B*,*C*_(long). In the case of pairwise distributions *ρ*_*rr*_ and *ρ*_*vv*_, the average is taken over pairs {(*x*_*i*_, *v*_*i*_), (*x*_*j*_, *v*_*j*_)} where *i* ranges over only the species in question and *j* ranges over the entire population.

Clusters 4 and 5 of **X**_*A*,*B*,*C*_(long) contain the remaining 31 *A* and *B* cells (3.1% of the total population); however, the learned forces in each cluster are still accurate: we find that Δf<2% for all forces and all clusters (row 3 of table 4). The validation error is high for cluster 5, reaching ΔV=35%, which indicates that the cells in cluster 5 have trajectories that are particularly sensitive to perturbations. Given the complexity of the dynamics (one can observe sharp turns taken by cells in the bottom two plots of figure 12), trajectories cannot be expected to remain close for all time, and in this case the validation error (3.19) may be too strong a metric.^10^ It is thus remarkable that the aggregate models for clusters 1, 2 and 3 produce accurate learned trajectories.

**Figure 17.**
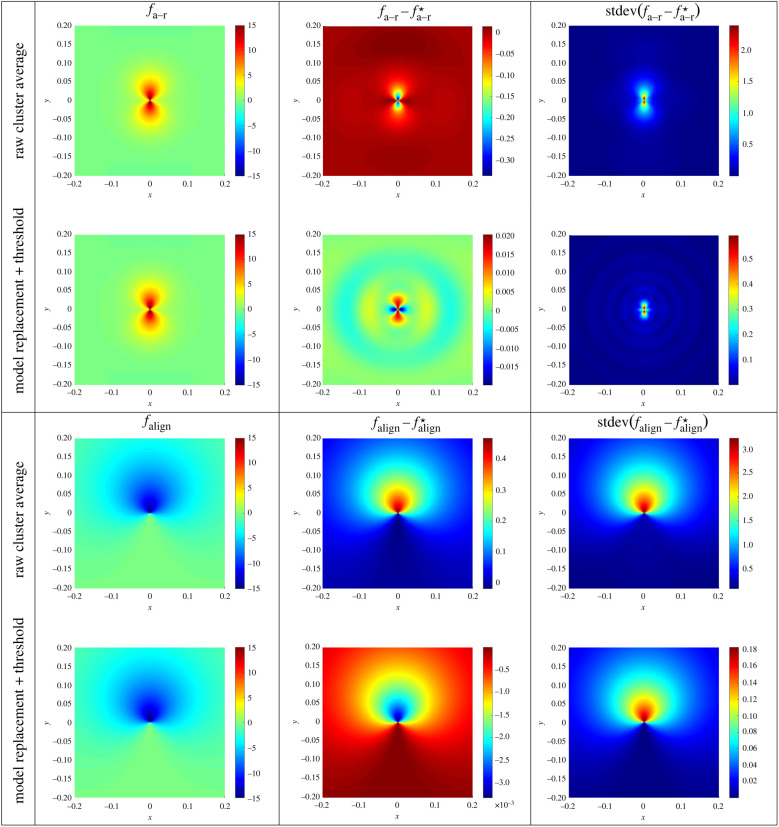
Statistical comparison of learned forces *f*_a−r_ and *f*_align_ from cluster 3 of the **X**_*A*,*B*,*C*_(long) data with and without the model replacement step followed by a final round of thresholding as in equation (3.16). Left to right: learned force, difference between true and learned force, and pointwise standard deviation of the individual models. To compute standard deviations for the model replacement + threshold case, individual models are modified to have the same coefficient support as the aggregate coefficients $\bar{w}$. While each learned force agrees well with the true force in the ‘eyeball norm’ (left column, compare with true forces in figure 2), it is clear from the middle and right columns that model replacement and a final thresholding step significantly reduce pointwise error and variance.

For a more in-depth statistical view of the algorithm for **X**_*A*,*B*,*C*_(long), in figure 17 we depict the average pointwise error and variance of the learned forces *f*_a−r_ and *f*_align_ across all individual learned models for cells in cluster 3. Moreover, we compare the effects of computing the aggregate model (given by the coefficients $\bar{w}$) as a raw cluster average versus first performing the model replacement step and then thresholding the final coefficients. For each force, we see that both methods produce satisfactory models to the eye, yet examining the pointwise error and variance reveals that the model replacement + thresholding step reduces errors by orders of magnitude.

## 5. Discussion

We have introduced a method for performing a combined classification and model selection task relevant to heterogeneous systems of autonomous agents. Specifically, we have shown that learning an ensemble of interacting particle models (one for each agent) allows iterative classification of agents into species according to their forward simulation accuracy. This is surprising due to the limited information carried in a single trajectory. Fortunately, the validation errors empirically approximate a log-normal distribution, hence Gaussian mixture model classification arises as the appropriate tool for identifying species membership.

Computational feasibility of this approach is grounded in the parallel nature of both the learning algorithm and the simulation component. Learning each single-trajectory model is cheap, with linear systems of size *m* × *n* with *m* and *n* not exceeding several hundreds (see table 7 for wall times). In the simulation step, each trajectory is validated separately and in parallel, and neighbour interactions are computed using the measurement data itself, resulting in O(N) pairwise interaction computations per time step instead of an $O(N^{2})$ full simulation.^11^

As previously mentioned, this approach is widely applicable to heterogeneous interacting particle systems, but there are many opportunities for extension. New techniques will need to be developed to effectively model particles which switch behaviours over time, or for non-conserved particle number (due to e.g. cell division or death). Multiple species which share force modes but vary in their magnitudes will also be challenging to identify using the proposed method. It is also possible that for highly diffusive particles the validation metric (3.19) is too restrictive, and a purely position-based metric should be used to classify species.

Lastly, several aspects of this approach deserve a more technical analysis, which may lead to improvements. We aim in future work to undertake more rigorous study of each component of the algorithm as outlined below.

### 5.1. Learning single-cell models

(I) **Information content**: It would be beneficial to quantify the information content in each cell trajectory, possibly eliminating trajectories that do not provide sufficient information. Model replacement, as outlined in §3.2, is an initial step in this direction. We saw in the experiments **X**_*A*,*B*_ and **X**_*A*,*B*,*C*_ that increasing the length of the trajectory leads to better classification when two species exhibit similar dynamics. It may be possible to use existing techniques, such as force matching, to identify highly informative cells from forces magnitudes, neighbour distributions, etc.
(II) **Noisy trajectories**: To focus on the classification task and equation learning methodology, we have neglected to add noise to trajectories in this work. However, it is reasonable to anticipate that measurement noise will be filtered out by the weak form as previously demonstrated on ODEs [62], PDEs [63] and first-order IPS [12]. We leave full examination of the robustness to both intrinsic (e.g. Brownian) and extrinsic (e.g. measurement) noise to future work.
(III) **Model library**: We chose the force bases and constraints to reflect physical properties, namely short-distance repulsion, long-distance decay, negative alignment, and negative drag. Directional modes enforce bilateral symmetry, and are low order (monopole, dipole, quadrupole). These can easily be adapted to incorporate other known information; moreover, the bases themselves may be adapted to the data (note this is partially done, using the neighbour distance distribution *ρ*_*rr*_ to restrict the range of interactions). One major assumption here is that there is no propulsion force, that energy is increased only through anisotropic interactions with neighbours. It would be interesting to examine whether this assumption holds true.
(IV) **Regression approach**: We employ modified sequential thresholding, which looks for an overall sparse solution, although we threshold *only* on the term magnitude ||**G**_*j*_**w**_*j*_|| and not that raw coefficient **w**_*j*_. This in particular allows *f*_a−r_, *f*_align_ and *f*_drag_ to have equal opportunity to enter the model despite different scales and bases used. The effect is that the resulting model is sparse in the *force modes* (as described in §3.3), while each force mode may have many components (in fact *f*_a−r_ is usually not sparse on a given directional mode). It may be more appropriate to use a group sparsity-enforcing method, such as constrained group LASSO. In general, this lies at the intersection of approximation and selection, where sparse selection is required to select the correct modes; however, the force content in each mode requires approximation. Explorations of the appropriate balance between selection and approximation would be valuable.

### 5.2. Cluster and aggregate

(I) Here we cluster models based on the directional modes present. This can easily be extended to the full pattern of non-zero elements in the model vector $\hat{w}$, although this depends on the number of resulting clusters.
(II) We have used a simple uniformly weighted average (3.16) to aggregate models; however, the use of model replacement (see §3.2) implies that the average is implicitly weighted according to *model generalizability*. Results may be improved if other criteria (e.g. information criteria) are incorporated into the weighted average, or if the median is taken instead of the mean.
(III) In the examples above, the aggregate model is used as the final model for the give class. Instead, one could further refine the model by performing an additional regression combining all data from the identified species.
(IV) Each cell experiences a different total number and duration of interactions with other cells. Accordingly, the models identified for each cell have varying levels of reliability, depending on the amount of information acquired to inform the model. This necessitated the development of our ad hoc classification scheme as we were unable to identify a suitable approach for sorting models with varying degrees of trustworthiness.

### 5.3. Validate and classify

In practice, the validation errors can easily be checked to satisfy lognormalcy *a posteriori*. If this is not satisfied, it may not be straightforward to cluster based on the validation error. In particular, chaotic trajectories cannot be expected to achieve a low validation error, in which case another metric is needed. In this case, it is reasonable to require that trajectories to be long enough to compute statistics. We aim to investigate the requirements for performing classification with chaotic interacting particles in future work.

**Table 5.** Summary of notation used throughout.

symbol	definition	location	value used here
C	set of model clusters based on force modes	§3.3	—
$\bar{C}$	cluster with the most members	§3.4	—
(**C**, **d**)	linear inequality constraint system	§A.3	equation (2.2)–(2.4)
(*ε*_gmm_, *δ*_gmm_)	halt classification if $P(VE<\epsilon_{\mathrm{gmm}})\geq 1-\delta_{\mathrm{gmm}}$	§3.7	(0.05, 0.01)
(*f*_a−r_, *f*_align_, *f*_drag_)_ℓ_	forces obeyed by cells in species ℓ	§§2.2–2.4	—
$f_{\mathrm{force}}^{\left(i\right)}$	directional force modes (force ∈ {a−r, align, drag})	§3.3	equation (3.15)
(**G**^(*i*)^, **b**^(*i*)^)	WSINDy linear system for learning model for cell *i*	equation (3.4)	—
*K*	number of neighbour cells chosen for model replacement	§3.2	32
*L*	number of time steps in data	§3.1	—
*L*′	number of time steps chosen for validation	§3.5	0.25*L*
λ	set of sparsity thresholds to sweep over	§3.1.3	(10^−4^, …, 1)
*λ*_log_	small threshold applied to abs. val of $\bar{w}$	§3.4	10^−4^
M	set of single-cell models	§3	—
$\bar{\;M}$	model associated with coefficients $\bar{w}$	§3.4	—
$\bar{\;M}(x,v,X,V)$	total force on (*x*, *v*) from neighbours (*X*, *V*) using model $\bar{\;M}$	§3.5	—
*N*	number of focal cells selected for learning	§3	*N*_tot_
*N*_tot_	total number of cells in the population	§2	—
*N*′_min_	minimum allowable number of cells in a species	§3.7	2
*n*_gmm_	number of 2-GMM fits to average over	§A.4	20
*p*_nf_	probability used to determine near-field radius *r*_nf_ for *f*_a−r_	§2.2	0.001
$\Phi ={\left\{\phi_{q}\right\}}_{1\leq q\leq Q}$	test functions to compute weak time derivatives	equation (3.5)	equations (3.5)
*r*_ff_	far-field radius, above which *f*_a−r_ is attractive	§2.2	1
*s*	independent variable for cell speed	§2	—
$\bar{S}$	species identified as obeying the model $\bar{\;M}$	§3.6	—
*S*_max_	maximum allowable number of species	§3.7	10
$\left(\tau ,\hat{\tau }\right)$	test function hyperparameters	§3.1.1	(10^−10^, 3)
*θ*_*ij*_	angle between *v*_*i*_ and *x*_*i*_ − *x*_*j*_	§2.1	—
$\Theta =(F_{a-r},F_{\mathrm{align}},F_{\mathrm{drag}})$	library of force functions for learning	§§3.1.1, 3.1.2	equations (3.6)–(3.9)
Δ*t*	time step of data	§3.1.1	—
Δ*t*′	time step for validation simulations	§3.5	2^−5^Δ*t*
Δ*V*_*i*_	validation error of cell *i*	equation (3.19)	equation (3.19)
*VE*	set of validation errors	§3.5	—
$w^{⋆}$	true model coefficients	§3.1.1	—
${\hat{w}}^{\left(i\right)}$	learned model coefficients for cell *i*	equation (3.10)	—
$\bar{w}$	coefficients obtained from averaging the models in cluster	equation (3.16)	—
(*X*, *V*)	cell population position and velocity	§2	—
(*x*_*i*_, *v*_*i*_)	position and velocity of cell *i*	§2	—
(**X**, **V**)	position and velocity time-series data	§3.1	—
(**x**_*i*_, **v**_*i*_)	position and velocity of cell *i* in time-series data	§3.1	—
$\left({\bar{x}}_{i},{\bar{v}}_{i}\right)$	cell *i* validation data simulated with model $\bar{M}$	equations (3.17), (3.18)	—
(**X**^′*i*^, **V**^′*i*^)	cell data with *i*th cell removed	§3.5	—

## Figures and Tables

**Figure 13.**
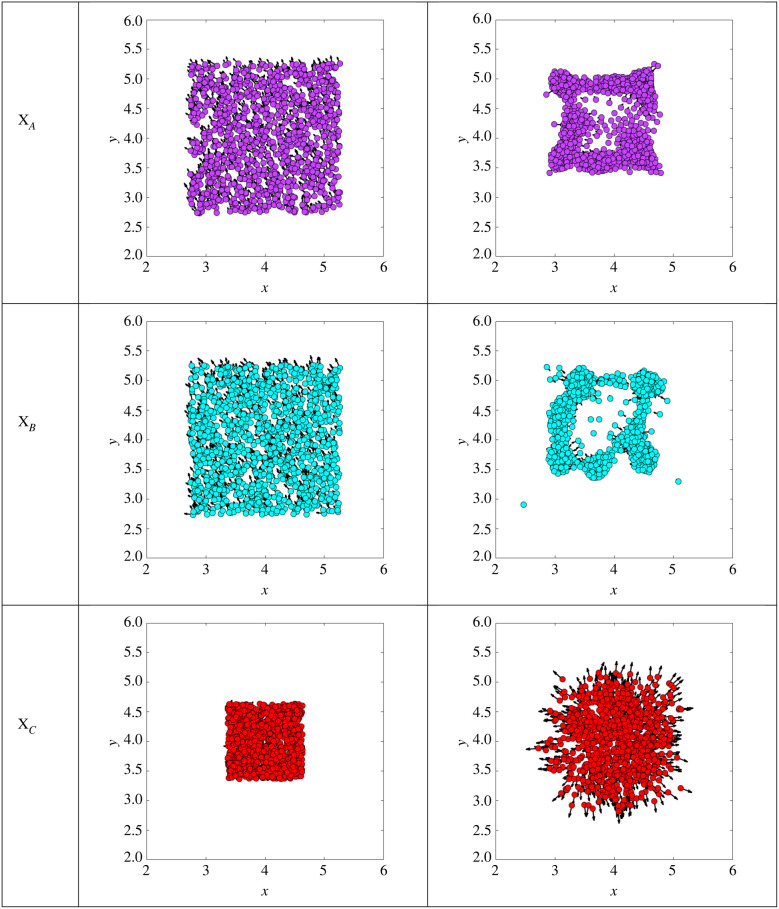
Plots of artificial data for homogeneous experiments at initial (left) and final (right) times.

**Figure 14.**
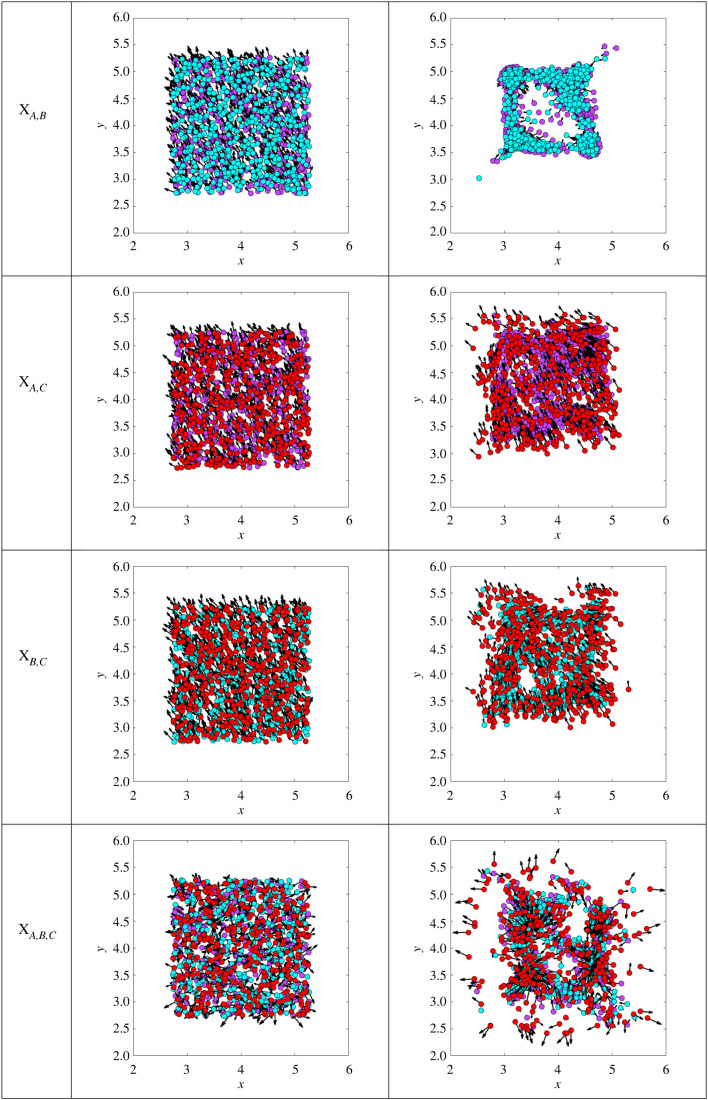
Similar to figure 13 but for heterogeneous experiments. Species are colour-coded as in figure 13.

## Data Availability

All software used to generate the results in this work is available at this repository: https://github.com/MathBioCU/WSINDy_CellCluster.git.

## Endnotes

1 See table 5 for a complete list of notations used.
2 For the artificial data used here, the set of focal cells is the entire population (*N* = *N*_tot_); however, in practice imperfectly tracked cells should be removed from the set of focal cells.
3 In order to isolate the classification task, in this work we do not examine noisy data other than numerical errors arising from coarse time-sampling.
4 In some cases, we can use weak-form integration to eliminate this step but we do not pursue this in the current work.
5 Details on this implementation, in particular **C** and **d**, are given in appendix A.3.
6 To limit the computational overhead, we only compute these KL divergences to cell *i*’s nearest 200 neighbours in the Euclidean sense.
7 As explored in [66], in some cases it may be more appropriate to use the coefficient median, or take a weighted average; however, the model replacement step already induces a weighted average. We leave these concerns for future work.
8 By some abuse of notation, $\bar{M}(x,v,X,V)$ is used to denote the instantaneous force on a particle (*x*, *v*) from neighbouring cells (*X*, *V*) under model $\bar{M}$
9 See table 7 for simulation wall times.
10 We discuss this further in the conclusion but leave a complete investigation to future work.
11 We are not aware of other simulation approaches in the context of interacting particles that use the measurement data for direct calculation of the forces.
12 In fact each of these parameters was chosen merely to reach a heuristic level of sufficiency. For example, we let Δ*t*′/Δ*t* = 2^−5^ because Δ*t*′/Δ*t* = 2^−6^ did not improve simulation accuracy, and we chose *K* = 32 to provide a sufficiently large neighbourhood of cells for model replacement. We did not test whether Δ*t*′/Δ*t* = 2^−5^ and/or *K* = 16 would also be sufficient, although these would provide computational savings and thus are worth examining in a future work.
13 In the case of Brownian cells, one modification could be to estimate the strength of the Brownian motion and validate models by averaging over multiple forward simulations of the corresponding stochastic differential equation. We leave this approach to a future work.

## Appendix A

**A.1. Notation, algorithm hyperparameters and wall times**

In table 5, we include notation used throughout the article along with locations in the text where each given symbol first appears and values of hyperparameters used in examples where applicable (last column). While the list of hyperparameters is quite long (rows of table 5 with entries in last column, 18 in total), many can be taken sufficiently large (*N*, *K*, *S*_max_, *n*_gmm_), sufficiently small (Δ*t*′, *λ*_log_, *N*′_min_), or sufficiently dense (λ), depending purely on available computational resources.^12^ The family of test functions Φ given by (3.5) has been demonstrated to work in a wide range of scenarios [12,62,63], and so may be taken as a fixed hyperparameter with minor tuning of the test function hyperparameters $\left(\tau ,\hat{\tau }\right)$, although it is possible that new application areas will require a different choice of test function class.

**Table 6.** Hyperparameters used for each example.

experiment	*m*	*p*	*r*_nf_	*r*_ff_
**X**_*A*_	38	8	0.0497	1
**X**_*B*_	38	8	0.0365	1
**X**_*C*_	32	9	0.0219	1
**X**_*A*,*C*_	35	9	0.0494	1
**X**_*B*,*C*_	35	9	0.0499	1
**X**_*A*,*B*_	38	8	0.0365	1
**X**_*A*,*B*_(long)	31	9	0.0219	1
**X**_*A*,*B*,*C*_	38	8	0.0569	1
**X**_*A*,*B*,*C*_(long)	31	9	0.0253	1

The remaining hyperparameters are problem-dependent. The force library Θ, along with constraints (**C**, **d**, which depend on *p*_nf_, *r*_ff_) may be altered to include additional knowledge of the possible force modes present along with their constraints. The modes $f_{\mathrm{force}}^{\left(i\right)}$ over which models are partitioned (taken to be based on directional dependence in this work, see equation (3.15)) could be subdivided in other ways, as discussed in §3.3. The length *L*′ of time points over which to validate model simulations, along with the error function used to define the validation error Δ*V*_*i*_ (equation (3.19)) and hyperparameters related to the GMM fitting process (*ε*_gmm_, *δ*_gmm_), may need tuning in the case of severely noisy data or Brownian cells, or chaotic trajectories, where forward simulations may not remain accurate for long.^13^ We conjecture that each of these hyperparameters may be learned from the given dataset (plus available domain knowledge), although we leave derivation of a direct map from data to hyperparameters to future work.

Several hyperparameter choices for the examples above which were not covered in §§3–4 are listed in table 6.

We include wall times for the main components of the algorithm in table 7, recorded in Matlab using an AMD Ryzen 7 pro 4750u processor. Detailed description of the implementation with respect to possible parallelism is included in the figure caption.

**Table 7.** Wall times for main components of one iteration of the algorithm, recorded for the **X**_*A*,*B*,*C*_(long) experiment (*N*_tot_ = 1000 cells and *L* = 400 time points). Each component contains an inner iteration which may be trivially parallelized. The reported times are for one step of the respective inner iteration. Specifically, it takes 5–10 s to learn each model $M_{i}$, while computation time for the full set of models $M=\{M_{1},\;\ldots ,M_{N}\}$ depends on the number of available CPUs. To form the model clusters $C=\{C_{1},\;\ldots C_{r}\}$, find the largest cluster $\bar{C}$, and compute the averaged model $\bar{M}$, forward simulations of each model $M_{i}$ (see §3.2) are performed which take 10–30 s (depending on the complexity of $M_{i}$). Similarly, computation of each Δ*V*_*i*_ requires one forward simulation (10–30 s). Lastly, to identify $\bar{S}$, it takes less than 1 second to perform GMM classification of log_10_(VE), and the results of 20 rounds of classification are averaged. If full parallelization is available, the wall time is less than 2 min per outer iteration, or less than 2*S* minutes in total, where *S* is the number of identified species. For comparison, the cost of generating the data for **X**_*A*,*B*,*C*_(long) takes 5–6 h.

$x_{i}\to M_{i}$	$M\to (\bar{C},$	$(x_{i},$	$VE\to \bar{S}$
5–10 s	10–30 s	10–30 s	<1 s

**A.2. Simulation details**

All example data were generated from the exact models using forward Euler with a time step of Δ*t** ≈ 0.00042 up until a final time of *T* ≈ 26. The time series was then coarsened to a resolution of Δ*t* = 0.13, resulting in a total of *L* = 200 time points for learning. Experiments **X**_*A*,*B*_(long) and **X**_*A*,*B*,*C*_(long) are simply extensions of **X**_*A*,*B*_ or **X**_*A*,*B*,*C*_ by an additional 200 time points at the resolution Δ*t* = 0.13. Initial positions were generated using Latin hypercube sampling in a box of side length 2. Initial velocities were drawn i.i.d. from a Gaussian distribution, where for **X**_*A*,*B*,*C*_, **X**_*A*,*B*,*C*_(long) and **X**_*C*_ we chose a mean velocity of $\bar{v}=(0,0)$ and covariance $\Sigma =0.0025I_{2}$, and for all other experiments we used $\bar{v}=(-0.02,0.035)$, $\Sigma =\left[\begin{array}{cc} 0.0014 & 0.0005\\ 0.0005 & 0.0012 \end{array}\right]$. The two sets of initial conditions represent different migratory stages, correlated or uncorrelated motion. See figures 13 and 14 for initial and final (time step 200) states of each experiment.

In figures 15 and 16, we include statistical information for species *A*, *B* and *C* in homogeneous experiments as well as the heterogeneous experiment **X**_*A*,*B*,*C*_(long). In each figure, the top row contains averages of the distributions used to select validation cells in the model replacement step (equations (3.12)–(3.14)). The bottom rows contains average polarization and angular momentum, respectively, defined as

$$\begin{array}{rl} P(t) & =\left|\frac{\sum_{i\in I_{S}}v_{i}(t)}{\sum_{i\in I_{S}}|v_{i}(t)|}\right|\;\mathrm{and}\;\\ M_{\mathrm{ang}}(t) & =\left|\frac{\sum_{i\in I_{S}}r_{i}(t)\times v_{i}(t)}{\sum_{i\in I_{S}}|r_{i}(t)\|v_{i}(t)|}\right| \end{array}\;(A\;1)$$

where *I*_*S*_ represents all cells in species *S* ∈ {*A*, *B*, *C*} and *r*_*i*_(*t*) = *x*_*i*_(*t*) − *x*_*c*_(*t*) where *x*_*c*_ is the center of mass of the entire population. The order parameters *P*(*t*) and *M*_ang_(*t*) are used to characterize phenotypes such as milling and spreading behaviour (e.g. [72]); however, they do not appear to indicate strong agreement with either phenotype in our datasets. Species *A* and *B* have nearly identical speed distributions (top right of each figure), in contrast to species *C*, with cells travelling slower and exhibiting stronger alignment (top middle of each figure). Notice that the algorithm is able to distinguish between *A* and *B* cells despite overall statistical similarity between the two species.

**A.3. Constrained sparse regression**

The constrained sequential thresholding algorithm requires solving at each thresholding iteration ℓ a linearly constrained quadratic program of the form

w(ℓ+1)=arg minws.t.Cw≤dsupp(w)⊂I(ℓ)⁡‖Gw−b22‖(A 2)

where $I^{\left(\ell \right)}$ is the set of coefficients of **w**^(ℓ)^ satisfying (3.11). The constraint system **C****w** ≤ **d** has the following four components.

(i) *f*_a−r_ ≥ 0 when 0 ≤ *r* < *r*_nf_: for the near-field repulsion of *f*_a−r_ we discretize the region $\left\{(r,\theta )\;:\;0\leq r<r_{\mathrm{nf}},\;\theta \in [0,2\pi )\right\}$ choosing five equally spaced points in *r* from 10^−6^ to *r*_nf_ and five equally spaced points in *θ* from 0 to *π*. Evaluating each of the basis function for *f*_a−r_ at this grid results in a constraint system **C**_a−*r*,*nf*_**w**_a−*r*_ ≤ **0** of dimension 25 × *J*_a−*r*_ where *J*_a−*r*_ is the number of basis functions used to approximate *f*_a−r_ and **w**_a−*r*_ is the restriction of **w** to coefficients of *f*_a−r_.
(ii) *f*_a−r_ ≤ 0 when *r* ≥ *r*_ff_: similarly for the far-field region, we choose 10 equally spaced points in *r* from *r*_ff_ to *r*_max_, where *r*_max_ is the maximum observed neighbour–neighbour distance in the simulation, and *θ* over the same points as previous. This results in a constraint system **C**_a−*r*,*ff*_**w**_a−*r*_ ≤ **0** of dimensions 50 × *J*_a−*r*_.
(iii) *f*_align_ ≤ 0: since the basis is positive, the constrain system here is simply $I_{J_{\mathrm{align}}}w_{\mathrm{align}}\leq 0$, where **I**_*n*_ indicates the identity on $R^{n}$.
(iv) *f*_drag_ ≤ 0: similarly the basis is positive, so the constraint system is $I_{J_{\mathrm{drag}}}w_{\mathrm{drag}}\leq 0$.

Altogether we get **d** = **0** and

$$C=\left[\begin{array}{ccc} C_{a-r,nf} & 0 & 0\\ C_{a-r,ff} & 0 & 0\\ 0 & I_{J_{\mathrm{align}}} & 0\\ 0 & 0 & I_{J_{\mathrm{drag}}} \end{array}\right]$$

We use Matlab's quadprog with constraint tolerance 10^−10^ and maximum iterations set to 1000. Note that since **d** = **0**, we do not lose feasibility during the thresholding step, which is possible in general. However, it is possible to arrive at the zero solution. This further necessitates the parameter sweep over *λ* values to select an appropriate threshold.

**A.4. Gaussian mixture model classification**

Since the Gaussian mixture model (GMM) fitting is performed using the expectation–maximization algorithm with random conditions, we perform the GMM fitting for *n*_gmm_ = 20 trials and identify $\bar{S}$ as the cells that in more than half of the trials appear in the mixture with lowest error.

At some stage in the algorithm, all the remaining cells will be homogeneous. In this case, a two-species Gaussian is the wrong model. To account for this, we do an initial fit to a single Gaussian and compute its Bayesian information criterion (BIC). We accept the two-mixture GMM if the average BIC of all 20 trial GMM fits with two mixtures is lower than that of the single Gaussian.

**A.5. Visualization tools**

In the repository https://github.com/MathBioCU/WSINDy_CellCluster.git we include the following scripts for visualization. (Note that the data can be downloaded at https://doi.org/10.5281/zenodo.6968448.)

— plot_true_forces.m: plots true forces, as in figure 2.
— visualize_trajectories.m: plots learned trajectories versus true trajectories, as in figures 8–12.
— plot_modelForce_stats.m: plots statistics related to individual models, as in figure 17.
— plot_gmm_script.m: plots Gaussian mixture models approximating the log-validation errors, as in figures 4–6.
— plot_individual_models.m: view subset of individual model forces (not included in the figures here).
